# Long-term gabapentin treatment impairs cognitive function in aged mice via tau hyperphosphorylation

**DOI:** 10.3389/fphar.2025.1616775

**Published:** 2025-09-03

**Authors:** Suyun Xia, Zerong You, Xinbo Wu, Jinsheng Yang, Shiyu Wang, Na Li, Jiajia Dai, Yuanlin Dong, Lucy Chen, Min Yan, Shiqian Shen, Zhongcong Xie, Jianren Mao

**Affiliations:** ^1^ Department of Anesthesiology, Second Affiliated Hospital of Zhejiang University School of Medicine, Hangzhou, China; ^2^ Department of Anesthesia, Critical Care and Pain Medicine, MGH Center for Translational Pain Research, Massachusetts General Hospital, Boston, MA, United States; ^3^ Department of Anesthesia, Critical Care and Pain Medicine, Massachusetts General Hospital, Shriners Hospital for Children, Boston, MA, United States; ^4^ Department of Orthopedics, Shanghai Tenth Hospital, Tongji University School of Medicine, Shanghai, China; ^5^ Department of Anesthesiology, 920th Hospital of Joint Logistic Support Force, Kunming, China

**Keywords:** aged mice, cognitive dysfunction, gabapentin (GBP), tau, Sirt1, CaMKIIα

## Abstract

**Introduction:**

Gabapentin (GBP) is widely prescribed to older patients for pain management. Recent clinical studies highlight that GBP adversely affect cognitive function in older patients. GBP binds to the α2δ1 subunit of L-type voltage-gated Ca^2+^ channels to inhibit Ca^2+^ channel current. It is being increasingly recognized that GBP affects neuronal activity in multifaceted ways. However, the molecular mechanism underlying GBP’s impact on cognitive function in older subjects remains unelucidated.

**Methods:**

Aged mice (18-month-old, female) were subjected to spared nerve injury (SNI) or sham surgery and treated with GBP for 60 days. Learning and memory were assessed using novel object recognition (NOR) test and contextual and cued fear conditioning test (FCT). Adeno-associated viral vector (AAV) was used for gene overexpression in the brain. Brain tissue was analyzed by Western blot, qRT-PCR, and protein activity assay.

**Results:**

Long-term GBP treatment impaired learning and memory in aged mice with or without nerve injury-induced pain as GBP-treated aged mice had lower novel object recognition index in NOR test and shorter freezing time in FCT, respectively. In the hippocampus of GBP-treated mice, increased levels of p-tau (S416) and p-tau (S262) were observed, together with increased CaMKIIα and decreased Sirt1 expression. AAV-mediated Sirt1 overexpression in the hippocampus or systemic administration of the Sirt1 activator resveratrol prevented cognitive impairment and tau hyperphosphorylation via enhancing Sirt1 activity in GBP-treated mice.

**Conclusion:**

Long-term GBP treatment is detrimental to cognitive function in aged mice. GBP suppressed Sirt1 expression, leading to elevated CaMKIIα level and hyperphosphorylation of tau, and boosting Sirt1 activity curbed the adverse effect of GBP on memory in aged mice.

## 1 Introduction

Gabapentin (GBP) is an FDA-approved drug for treatment of partial seizures, postherpetic neuralgia, and restless legs syndrome (Pauly et al., 2020; Johansen and Maust, 2024). GBP is also prescribed off-label for anxiety and neuropathic pain management (Johansen and Maust, 2024). GBP prescriptions increased by roughly 170% from 2009 to 2016 (Pauly et al., 2020) and continue to increase (Johansen and Maust, 2024). More than 50% of GBP prescriptions are written for patients over 65 years old for pain management (Johansen and Maust, 2024), in part due to its favorable pharmacokinetic profile (Striano and Striano, 2008). Clinical GBP therapy for pain management often lasts for months or even years (Mao and Chen, 2000; Bonnet and Scherbaum, 2017; Mersfelder and Nichols, 2016; Smith et al., 2012) and older patients are often prescribed with a high dosage (Fleet et al., 2018; Moore et al., 2014). Recent clinical studies have highlighted the harmful effects of GBP on cognitive function in older patients besides the known side effects of GBP such as somnolence and disorientation. Among older adults with initially normal cognition, initiation of GBP treatment induced impairment of cognitive function (Oh et al., 2022) and perioperative GBP use is associated with increased risk of delirium among older adults after major surgery (Park et al., 2022).

GBP is an anti-seizure and anti-nociceptive agent, the mechanism of action likely involves its inhibition of calcium currents via binding to the α2δ1 subunit of L-type voltage-gated Ca^2+^ channels (Hendrich et al., 2008; Gee et al., 1996). Rodent studies have shown that GBP affects cognitive function (Gregoire et al., 2012; Celikyurt et al., 2011), and other neuronal activities in multifaceted ways. GBP antagonizes thrombospondin binding to the α2δ1 subunit and strongly inhibits excitatory synapse formation in neonate mice (Eroglu et al., 2009). GBP enhances the expression levels of δGABAA receptors and increases a tonic inhibitory conductance in neurons (Yu et al., 2019). GBP activates locus coeruleus (LC) neurons to induce norepinephrine release in the prefrontal cortex (Hayashida et al., 2008). However, the molecular mechanism as to how GBP affects cognitive function in older adults is unknown.

In the present study, we examined the impact of long-term GBP treatment on cognitive activity and investigated the underlying molecular mechanism in aged mice (18 months old). We found that GBP treatment impaired cognitive function in aged mice tested using novel object recognition test and contextual and cued fear conditioning test. In the hippocampus of mice treated with GBP, we observed elevated phosphorylation of tau protein at S416 and S262 sites, together with increased Ca^2+^/calmodulin-dependent kinase II alpha subunit (CaMKIIα) and decreased Sirt1 expression. Adeno-associated virus (AAV) vector mediated overexpression of Sirt1 in the hippocampus or systemic administration of Sirt1 activator resveratrol curbed GBP-induced cognitive impairments and suppressed tau phosphorylation. Taken together, our study revealed a Sirt1-CaMKIIα-tau signaling pathway underlying GBP-induced cognitive impairment in aged mice. Enhancing Sirt1 activity with resveratrol could be a potential remedy to curb this side effect of GBP.

## 2 Methods

### 2.1 Experimental animals

Female C57Bl/6J mice of 18 months of age (Jackson laboratory) were used in the study. All mice were maintained at MGH animal housing facility in a specific pathogen free (SPF) environment. The room temperatures were 19 °C–23 °C, the humidity was 40%–60%, and had a 12 h light/dark cycle. Three to four mice were housed in each ventilated cage. Food and water were provided *ad libitum*. Mice were randomly assigned to experimental groups. Gabapentin (100 mg/kg, Sigma-Aldrich, G154) and resveratrol (40 mg/kg, Fisher Scientific, R0071) were administered by intraperitoneal (i.p.) injection. Gabapentin was dissolved in saline (5 mg/mL), and resveratrol was dissolved in a vehicle solution (5% DMSO+30% PEG300 in water) to obtain 15 mg/mL solution. The respective dissolving solution was used as vehicle control. To reduce stress, animals received 1 day break after every 6 days of injection. The animal protocol was approved by Massachusetts General Hospital Institutional Animal Care and Use Committee.

### 2.2 Spared nerve injury

Spared nerve injury (SNI) was produced as previously described (Decosterd and Woolf, 2000). Briefly, a skin incision on the left thigh was made on a fully anesthetized mouse (1%–2% isoflurane in 100% O_2_) to expose the sciatic nerve. The common peroneal and tibial nerve branches were completely sectioned, leaving the sural nerve branch intact. Sham-operated mice were subjected to the same procedure without sectioning the nerves. The incision site was sutured, and mice were returned to home cages when fully recovered from anesthesia.

### 2.3 Behavioral test

All behavioral tests were carried out by the investigators who were blinded to experimental groups. Mice were habituated to the test environment for two consecutive days (30 min per day) prior to testing.

#### 2.3.1 von frey test

Mechanical allodynia was assessed using von Frey filaments (Decosterd and Woolf, 2000) (Sensory Evaluator Kit, Stoelting Co., Wood Dale, IL, United States). A single filament was applied to the plantar surface of a hind paw for five times with an interval of 5 s. The smallest filament that produced at least twice paw withdrawal was recorded as paw withdrawal threshold.

#### 2.3.2 Open field test (OFT)

Open field test (Kraeuter et al., 2019) was conducted in a plexiglass square box (57 × 57 × 50 cm). The mouse was allowed to run freely for 1 min, afterwards, the activity of the mouse was tracked for 5 min and analyzed by SMART video-tracking system (Panlab, Harvard Apparatus, MA, United States).

#### 2.3.3 Novel object recognition (NOR) test

NOR test includes a training and a testing session (Leger et al., 2013). During a 5-min training session, the mouse familiarized itself with two identical objects in a plexiglass box (34 × 17 × 17 cm). After a 10-min home cage stay, the mouse was tested for 5 min with one of the familiar objects replaced with a novel object. In both training and testing sessions, the time the mouse spent with each object was recorded. A recognition index (RI) for each animal was calculated as the ratio TN/(TF + TN) (TN: time spent with a novel object, TF: time spent with a familiar object).

#### 2.3.4 Contextual and cued fear conditioning test (FCT)

Contextual and cued fear conditioning test (FCT) was conducted using Stoelting™ Fear Conditioning System paired with ANY-Maze Behavioral Tracking Software. In training, the mouse was placed in the conditioning chamber (black/white stripes pattern) for 3 min prior to being subjected to a 2-Hz pulsating tone (80 dB, 3,600 Hz, 60 s). A mild foot shock (0.8 mA for 0.5 s) was applied immediately after the tone. Mice were tested at 3 and 7 days after training. On the test day, mice were subjected to both context test and cued test. In context test, the mouse stayed in the same chamber (black/white stripes pattern) for a total of 6 min without application of tone and foot shock. The amount of time the mouse demonstrated “freezing behavior” was tracked and recorded. Tone (cue) test was performed 2 h later. In tone test, the mouse stayed in a different chamber (black/white checkered pattern) for a total of 6 min. The same tone used during training was applied for the last 3 min without the foot shock, and the “freezing behavior” was tracked and recorded. The “freezing behavior” was defined as a completely immobile posture except for respiratory effort. Any-Maze setting: freezing on threshold: 10; freezing off threshold: 20; minimum freezing duration: 1 s (Stoeling Co.)

### 2.4 Hippocampal injection of AAV

To overexpress Sirt1 in the hippocampus, adeno-associated virus 9 (AAV9) vector carrying Sirt1 or eGFP was purchased from Vector Biolab (Malvern, PA, United States): AAV9-CMV-eGFP-2A-mSirt1 (Catalog number: 7000), AAV9-eGFP (Catalog number: 7007). The titer of viruses is 1 × 10^13^ gc/mL (genome copy/mL). The mice were anesthetized with isoflurane and secured in a stereotaxic frame (Kopf, Tujunga, CA, United States), and AAV was injected with the Nanoject III (Drummond Scientific Company, model 3-000-207) under sterile conditions. Bilateral injection was performed and holes of the size of the injection needle were drilled into the skull: 2.1 mm caudal to bregma, 1.5 mm ventral to pial surface, and 1.5 mm right of midline for right side injection and 1.5 mm left of midline for left side injection. Each side was injected with 1 μL of AAV vector (1 × 10^13^) at a rate of 0.2 μL/min.

### 2.5 Western blot analysis

The hippocampal tissue was harvested and stored at −80 °C until being processed. For Western blot analysis, tissue was homogenized in RIPA buffer (Cell Signaling 9806S) containing Protease inhibitor (Thermofisher A32953) and phosphatase inhibitor (1 mM sodium fluoride, 1 mM β- glycerophosphate, 0.5 mM sodium orthovanadate, and 0.5 mM sodium pyrophosphate). Protein (30 μg/lane) was separated on SDS-PAGE gel, transferred to PVDF membrane (Sigma Immobilon-P, IPVH00010), probed with antibodies, and detected using ECL substrates (SuperSignal West Pico Plus, ThermoFisher 34580) and X-ray films. PageRuler Plus Pretained protein standard (ThermoFisher 0026619) was used to estimate the protein size. X-ray films were scanned, and protein bands were quantified using ImageJ (NIH) (Schneider et al., 2012). p-Tau proteins were normalized to tau; Sirt1, CaMKIIα and tau were normalized to actin. In graphs, the values shown are relative to “sham + saline” or “AAV-eGFP + saline” or “saline + vehicle”. The antibodies used in this study are listed in Table 1.

**TABLE 1:** List of antibodies.

Antibody	Manufacturer	Catalogue no.	Dilution
tau mouse mAb	Sigma	T9450	1:500
Phospho-Tau Family Antibody Sampler Kit	Cell Signaling	96628	
Phospho Tau (Ser262) Rabbit polyclonal Ab	abcam	ab131354	1:1000
Phospho-Tau (Ser416) (D7U2P) Rabbit mAb	Cell Signaling	15013S	1:1000
Phospho-Tau (Ser396) (PHF13) Mouse mAb	Cell Signaling	9632	1:1000
Phospho-Tau (Ser404) (D2Z4G) Rabbit mAb	Cell Signaling	20194	1:1000
Phospho-Tau (Ser202) (D4H7E) Rabbit mAb	Cell Signaling	39357	1:1000
anti-CaM Kinase IIα (CaMKIIα) Rabbit Ab	Sigma	C6974	1:1000
anti-Sirt1 (B-10)	Santa Cruz	Sc-74504	1:1000
anti-CACNA2D1 (α2δ1) (A20) Mouse mAb	ThermoFisher	MA3-921	1:500
β-actin, Mouse mAb	Sigma	A1978	1:5,000
anti-mouse IgG-HRP	Santa Cruz	sc-2031	1:10,000
anti-rabbit IgG-HRP	Santa Cruz	sc-2357	1:10,000

### 2.6 Realtime quantitative PCR analysis

Total RNA was isolated from brain tissue using Trizol (Thermosfisher 15596026). cDNA was synthesized using ProtoScript First Strand cDNA Syn Kit (E6300L) from New England BioLabs (Ipswich, MA, USA). qPCR was preformed using TaqMan probes (CamKII2a: Mm00437967_m1; Gapdh: Mm99999915_g1) and TAQman universal MMIX II (Cat # 4440040) from Life Technologies (CA, United States) on a Quant 3 QuantStudio™ 3 Real-Time PCR system (Applied Biosystems). The data were analyzed by the ΔΔCt method. The mRNA level of CamKII2a was normalized to GAPDH, and the value in the graph is expressed as relative to “sham + saline”.

### 2.7 Protein activity assay

Hippocampal CaMKII activity was analyzed using CycLex CaM-kinase II assay kit (CY-1173) and CaM-Kinase II positive control (CY-E1173) (MBL International Corporation, Woburn, MA) according to the protocol provided by the manufacture. Sirt1 activity was analyzed using Sirt1 assay kit (Sigma, Cat # 1040) according to the protocol provided by the manufacturer.

### 2.8 Statistical analysis

Data were analyzed using GraphPad Prism 8 software and expressed as mean ± SEM. Unless specified, one-way ANOVA with Tukey’s multiple comparisons was used for the analysis. Two-way ANOVA was used for repeated measurement of paw withdrawal threshold in von Frey test. T-test was used to compare paw withdrawal threshold before and after GBP administration. The significance level was set at 0.05.

## 3 Results

### 3.1 Long-term GBP treatment impaired cognitive function in aged mice

**FIGURE 1:**
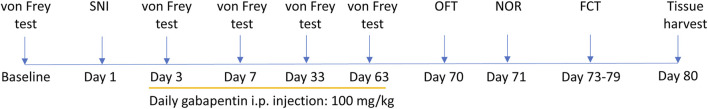
Experimental design. After assessing baseline paw withdraw threshold using von Frey test, mice were randomly assigned to experimental groups. Spared nerve injury (SNI) or sham surgery was performed on mice. Gabapentin (100 mg/kg/daily, i.p.) or saline treatment began on day 3 after surgery and lasted for 60 days. Von Frey test was conducted at 3, 7, 33, and 63 days after surgery before daily GBP or saline injection. Motor and cognitive functions were assessed starting at 7 days after the last dose of GBP or saline treatment. Brain tissues were harvested after completion of behavioral testing. OFT: open field test, NOR: novel object recognition test, FCT: contextual and cued fear conditioning test.

**FIGURE 2:**
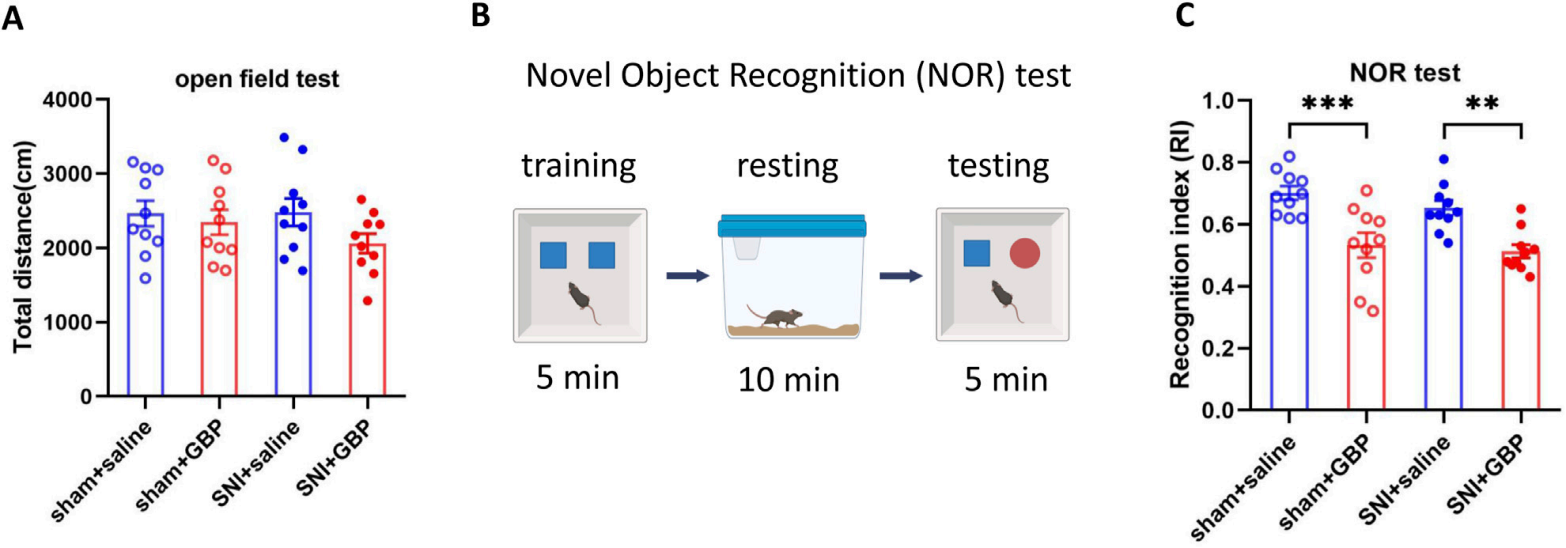
Long-term GBP treatment impaired short-term memory in aged mice. **(A)** Motor function was not affected by GBP treatment or nerve injury. In open field, the total distance traveled by the four groups of mice (sham + saline, sham + GBP, SNI + saline, and SNI + GBP) was not significantly different (one-way ANOVA, p = 0.27, n = 10/group). **(B)** Illustration of novel object recognition (NOR) test protocol. **(C)** In NOR test, GBP-treated mice had a lowered recognition index (RI) for the novel object than saline-treated mice (sham: saline vs. GBP, p = 0.0003. SNI: saline vs. GBP, p = 0.002. n = 10/group).

Aged (18-month-old) mice with SNI or sham surgery (sham) were treated with GBP (100 mg/kg/daily, i. p.) for ∼ 2 months. The dose of GBP was based on previous studies (Kusunose et al., 2010; Aydin et al., 2012; Cheng and Chiou, 2006). The experimental design is illustrated in Figure 1. At 7 days after administering the last dose of GBP or saline, OFT was performed to determine whether nerve injury and/or GBP treatment affected motor activity in aged mice. In the open field, the four groups of mice traveled similar total distance, suggesting that motor activity was not significantly affected in aged mice (one-way ANOVA, p = 0.27, n = 10/group) (Figure 2A).

Mice were subsequently subjected to NOR test to examine short-term memory (Cohen and Stackman, 2015) as illustrated in Figure 2B. In training phase, both saline- and GBP-treated sham or SNI mice spent an equal amount of time exploring two identical objects, and had a similar recognition index (RI) for both objects (Supplementary Figure S1). In testing phase, saline-treated mice (SNI or sham) spent more time exploring a novel object than GBP-treated sham or SNI mice did. Therfore, GBP-treated mice had a lower RI for the novel object, showing an impairment in recognition memory (Figure 2C) (one-way ANOVA with Tukey’s multiple comparisions test. sham + saline vs. sham + GBP: ***p < 0.001; SNI + saline vs. SNI + GBP: **p < 0.01, n = 10/group).

**FIGURE 3:**
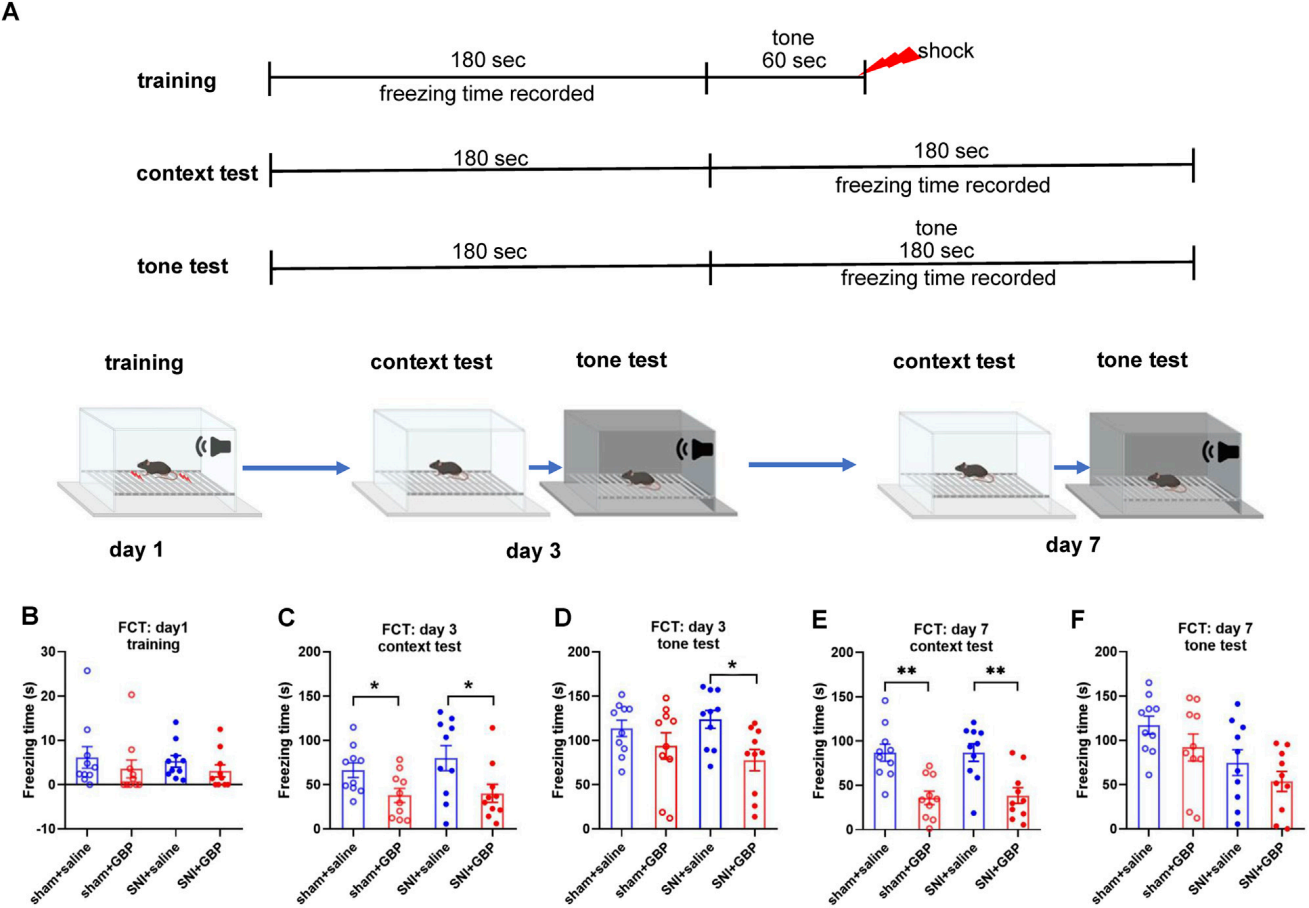
Long-term GBP treatment impaired associative learning and memory in aged mice. **(A)** Illustration of the protocol for contextual and cued fear conditioning test (FCT). **(B)** In training, mice did not differ significantly in freezing behavior (one-way ANOVA, p = 0.62, n = 10/group). **(C,D)** Test on day 3. In context test, GBP-treated sham or SNI mice had decreased freezing time than saline-treated mice (sham: saline vs. GBP, p = 0.03. SNI: saline vs. GBP, p = 0.02). In tone test, GBP-treated SNI mice had decreased freezing time than saline-treated SNI mice. (SNI: saline vs. GBP, p = 0.02). **(E,F)** Test on day 7. In context test, GBP-treated sham or SNI mice had decreased freezing time than saline-treated mice (saline vs. GBP, p < 0.01). In tone test, no differences were observed between saline and GBP treatment in sham or SNI mice.

Next, we performed FCT to assess associative fear learning and memory as depicted in Figure 3A. In training phase, the average freezing time was less than 10 s for all mice, and the four groups of mice did not differ significantly in freezing time (one-way ANOVA, p = 0.62, n = 10/group) (Figure 3B). Mice were tested on day 3 and day 7 for context- and tone-associated memory. In context test, GBP-treated mice had less freezing time than saline-treated mice (Figures 3C,E, day 3 and day 7; for sham or SNI groups, saline vs. GBP: *p < 0.05, **p < 0.01). In tone test, the freezing time was decreased in SNI mice when tested on day 3 (Figure 3D, SNI groups: saline vs. GBP, *p < 0.05), but not on day 7 (Figure 3F). We also compared the freezing time during the first 3 min in the chamber during context and tone test. Saline-treated mice had longer freezing time in the context test chamber (black/white stripes pattern) than in the tone test chamber (black/white checkered pattern), while GBP-treated mice did not exhibit differences (Supplementary Figures S2A,B), indicating that saline-treated mice could remember and distinguish the environment wherein they experienced shock, but GBP-treated mice could not. These data re-enforced our data presented in Figures 3B–E showing GBP-treatment induced cognitive impairments.

We assessed SNI-induced mechanical allodynia using von Frey test on day 3, 7, 33, and 63 prior to daily GBP administration. Mice with SNI exhibited prolonged mechanical allodynia compared to sham mice, as SNI mice had a lower threshold in response to von Frey fiber stimulation on the ipsilateral paw than sham mice (two-way ANOVA, p < 0.0001, n = 10/group (Supplementary Figure S3A). GBP treatment effectively attenuated nociception in SNI mice measured at 2 h after GBP administration (Supplementary Figure S3B), suggesting that signs of cognitive impairment in GBP-treated mice, as shown above, were not caused by SNI-induced pain.

Taken together, these data suggest that GBP treatment imparied cognitive function in aged mice with or without neuropathic pain.

### 3.2 Long-term GBP treatment increased tau phosphorylation in the hippocampus in aged mice

**FIGURE 4:**
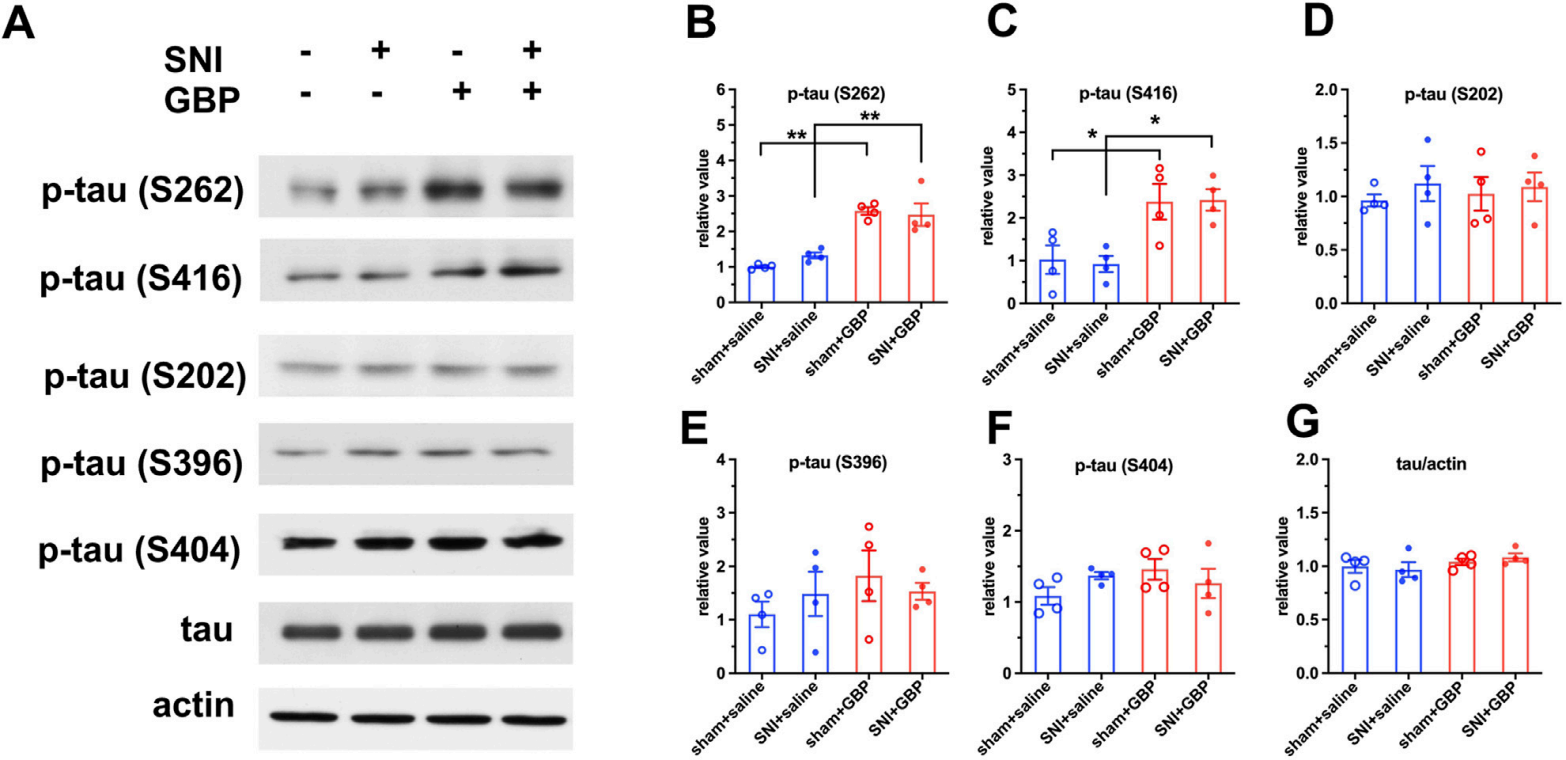
GBP treatment caused tau hyperphosphorylation in the hippocampus of aged mice. **(A)** Using Phospho-Tau Family Antibody Sampler Kit, the levels of total tau protein and phosphorylated tau at five sites were analyzed using Western blot analysis. **(B,C)** The levels of p-tau (S262) and p-tau (S416) were increased in GBP-treated sham and SNI mice (*P < 0.05, **P < 0.01, n = 4/group). **(D–G)** The levels of p-tau (S202), p-tau (S396), p-tau (S404), or total tau protein did not significantly differ between GBP- and saline-treated mice.

Tau proteins, a group of six isoforms, are microtubule-associated proteins and are highly expressed in neurons in the hippocampus and cortex (Mandelkow and Mandelkow, 2012; Binder et al., 1985; Jameson et al., 1980). We examined if GBP treatment caused abnormal tau phosphorylation, as dysregulation of tau phosphorylation is associated with cogntive impairment. We analyzed the hippocampus tissue using Phospho-Tau Family Antibody Sampler Kit and Western blot. GBP-treated mice had increased levels of p-tau (S262) and p-tau (S416) in the hippocampus compared to saline-treated mice (Figures 4A–C) (**p < 0.01, *p < 0.05, n = 4/group). The levels of total tau, p-tau (S202), p-tau (S396), and p-tau (S404) did not significantly differ between GBP and saline treatment groups (Figures 4D–H). Our observation indicates that hippocampal tau hyperphosphorylation is associated with GBP-induced congntive impariment in aged mice.

### 3.3 Long-term GBP treatment affected CaMKIIα and Sirt1 expression in the hippocampus of aged mice

**FIGURE 5:**
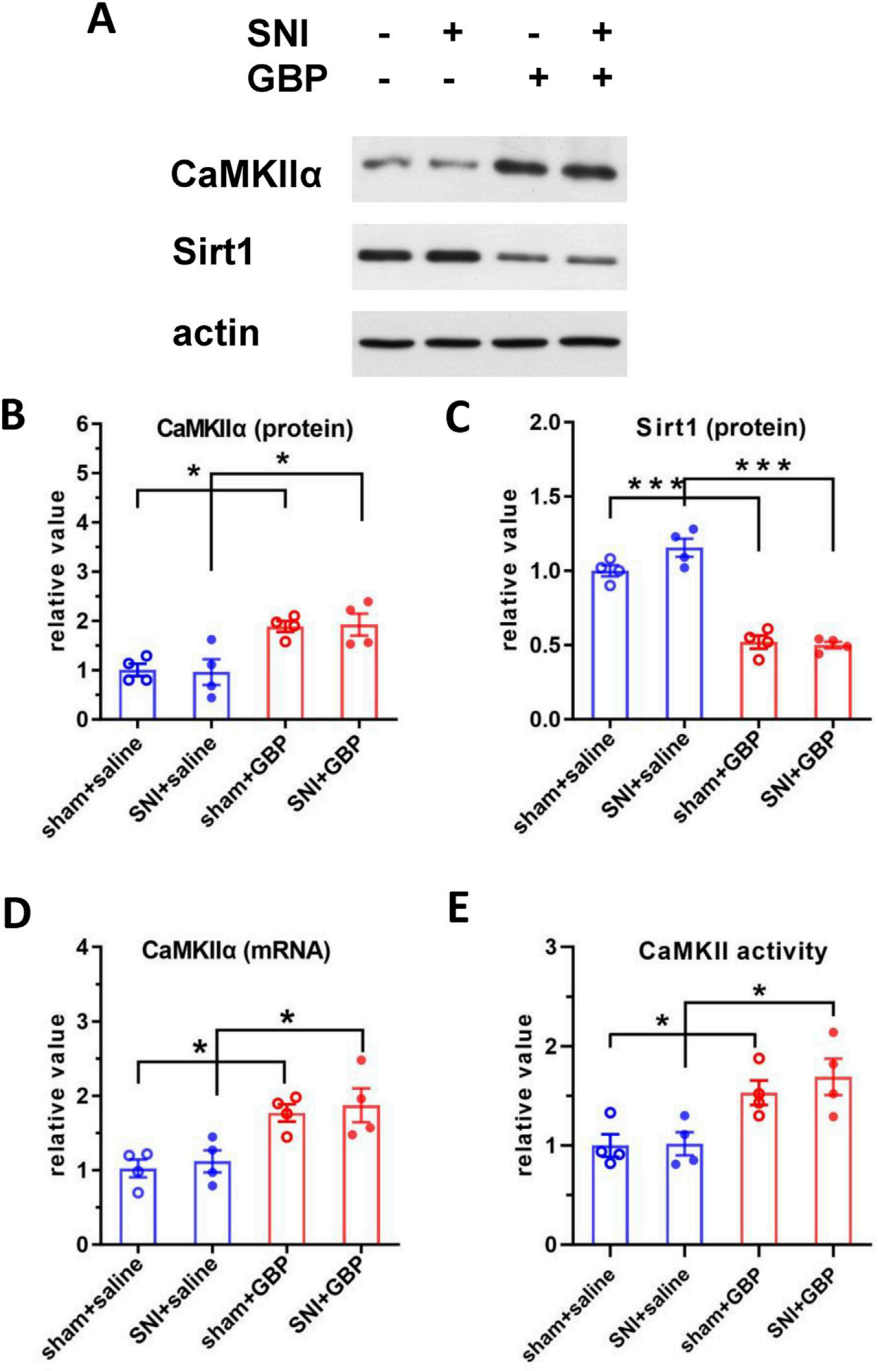
GBP treatment changed the expression of CaMKIIα and Sirt1 in the hippocampus of aged mice. **(A)** Western blot analysis of CaMKIIα and Sirt1. CaMKIIα protein level **(B)** was increased; Sirt1 protein level **(C)** was decreased in GBP-treated aged mice. CaMKIIα mRNA **(D)** and CaMKII kinase activity **(E)** were increased in GBP-treated aged mice. (*P < 0.05,***p < 0.001, n = 4/group).

Phosphoration of tau is regulated by several protein kinases. Ser/Thr protein kinase CaMKIIα preferentially phosphorylates Tau at S262 and S416 sites (Sironi et al., 1998; Yamamoto et al., 2005; Hector et al., 2020). CaMKIIα, a major isoform of CaMKII, is highly expressed in the hippocampus (Wang et al., 2013). Accordingly, we analyzed the expression levels and kinase activity of CaMKIIα. Western blot and q-RT-PCR analysis showed elevated CaMKIIα expression levels in the hippocampus of GBP-treated mice (Figures 5A,B,D). Moreover, the kinase activity of CaMKIIα in the hippocampus was also increased in GBP-treated mice (Figure 5E), which is consistent with the increase of CaMKIIα protein expression. To examine how GBP regulates CaMKIIα expression, we focused on Sirt1, a histone deacetylase, as Sirt1 transcriptionally regulates CaMKIIα expression via deacetylating histone H3 lysine 9 at the *CaMKIIα* promoter (Zhou et al., 2020). Sirt1 protein expression in the hippocampus was decreased in GBP-treated mice (Figure 5C). Taken together, GBP treatment led to differential expression of CaMKIIα and Sirt1 in the hippocampus of aged mice.

### 3.4 Overexpressing Sirt1 in the hippocampus attenuated GBP-induced cognitive impairment and tau phosphorylation in aged mice

**FIGURE 6:**
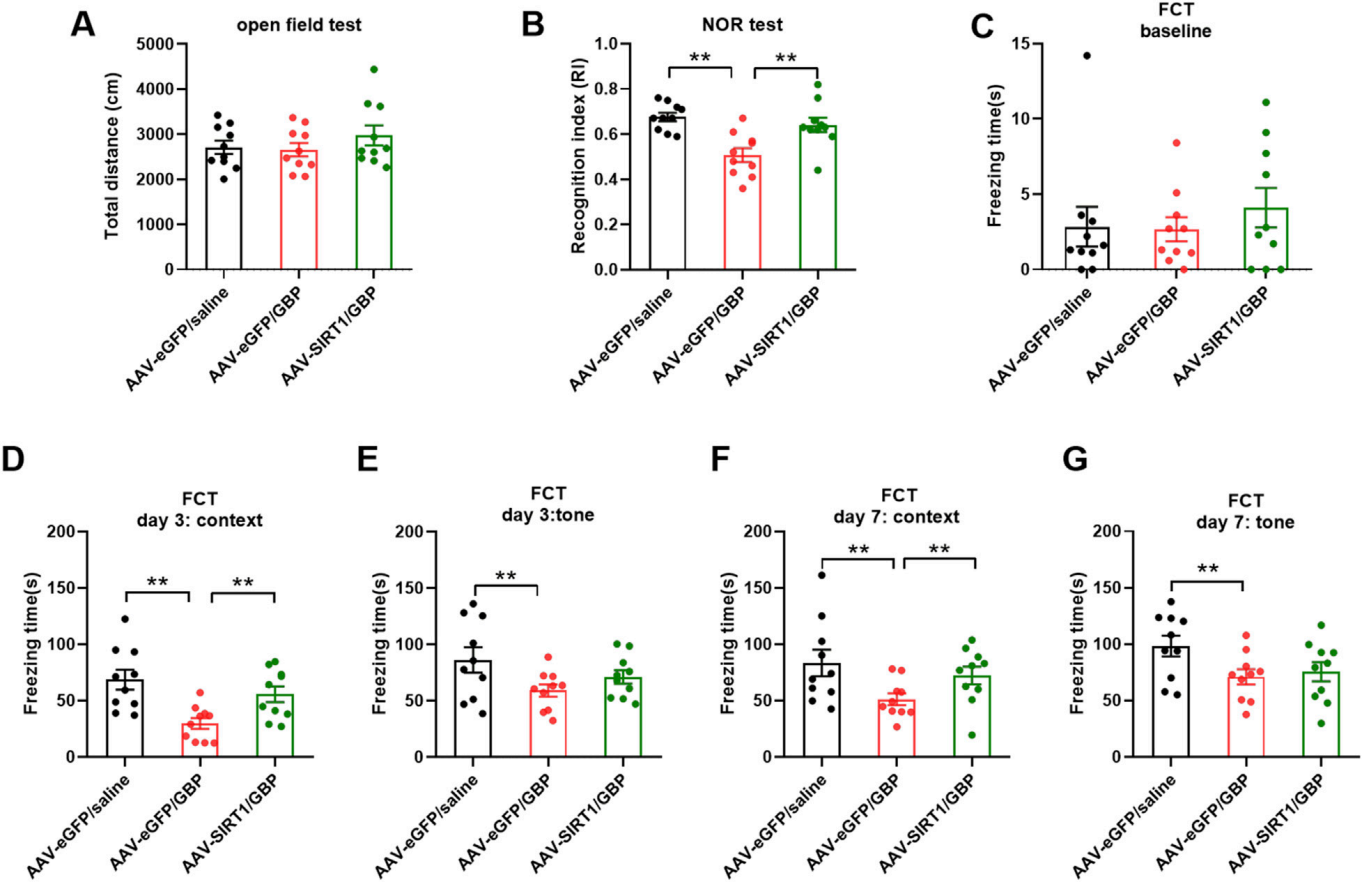
Sirt1 overexpression in the hippocampus prevented cognitive impairment in GBP-treated aged SNI mice. **(A)** In open field test, the total distance traveled by three groups of mice was similar (one-way ANOVA, p = 0.41). **(B)** In NOR test, mice overexpressing Sirt1 had increased recognition index (RI) for a novel object (**p < 0.01). **(C–G)** Contextual and cued fear conditioning test (FCT). **(C)** In training phase of FCT, baseline freezing time was not significantly different among three groups of mice (one-way ANOVA, p = 0.6). **(D–G)** Testing phase of FCT: tested on day 3 and 7 after training. In eGFP-expressing group, GBP-treated mice had shorter freezing time than saline-treated mice in both context and tone tests (**p < 0.01). For GBP treatment, mice with Sirt1 overexpression had longer freezing time than mice with eGFP expression in context test (**p < 0.01). (n = 10/group).

**FIGURE 7:**
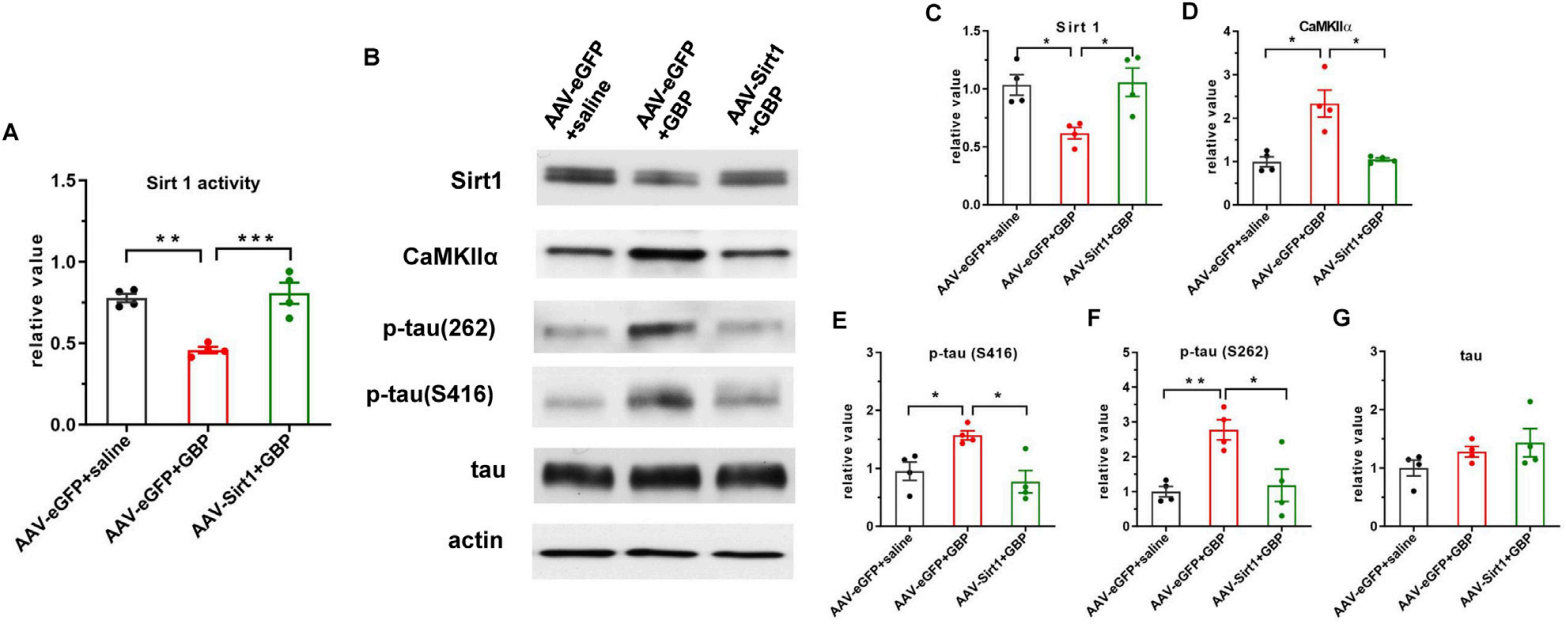
AAV-mediated Sirt1 overexpression in the hippocampus inhibited CaMKIIα expression and tau hyperphosphorylation in GBP-treated SNI aged mice. **(A)** Sirt1 overexpression restored Sirt1 activity in GBP-treated mice. **(B)** Western blot images of Sirt1, CaMKIIα, and tau proteins. **(C)** In eGFP expressing mice, Sirt1 protein levels were decreased by GBP treatment. **(D,E,F)** Overexpression of Sirt1 inhibited CaMKIIα protein levels, phosphorylation of p-tau (S416) and p-tau (216) in GBP-treated mice. **(G)** Total tau level was not affected by Sirt1 overexpression (*p < 0.05, **p < 0.01, ***p < 0.001, n = 4/group).

Since we identified that decreased Sirt1 level could contribute to tau pathology induced by GBP in aged mice, we examined if overexpression of Sirt1 rescued behavioral outcomes and tau pathology. Adeno-associated viral vector-mediated gene transfer and overexpression was used. AAV9-eGFP (control) or AAV9-eGFP-Sirt1 (10^13^ gc/mL, 1 µL/side, Vector Biolabs) were bilaterally infused into the hippocampus of 18-month-old (aged) mice. At 3 weeks after the AAV9 infusion, mice were subjected to SNI surgery, treated with saline or GBP for ∼ 2 months, and examined for behavioral outcomes as illustrated in Figure 1. AAV9 infusion did not affect motor function in mice (one-way ANOVA, p = 0.41, n = 10/group) (Figure 6A). Sirt1 or eGFP overexpression in the hippocampus did not affect SNI-induced nociception (Supplementary Figure S4). NOR and FCT tests showed that Sirt1 overexpression ameliorated cognitive impairment in GBP-treated mice (Figures 6B–G, GBP groups: eGFP vs. Sirt1, **p < 0.01, Supplementary Figures S2A,C, AAV-eGPF/saline and AAV-Sirt1/GBP: pre-context vs. pre-tone, *p < 0.05). Tissue analysis showed that AAV9-eGFP-Sirt1 infusion rescued Sirt1 expression levels and activity in GBP-treated mice, inhibited CaMKIIα expression and phosphorylation of p-tau (S416) and p-tau (S262) (Figure 7, ***p < 0.001, **p < 0.01, *p < 0.05, n = 4/group). As a control for AAV infusion, the expression of eGFP did not alter the adverse effect of GBP on cogntive function and tau phosphorylation in aged SNI mice (Figures 6, 7, AAV-eGFP groups: saline vs. GBP, **p < 0.01, *p < 0.05). Thus, overexpression of Sirt1 suppressed the adverse effects of GBP on cognitive activity and tau phosphorylation in aged mice.

### 3.5 Sirt1 activator resveratrol prevented cognitive impairments and tau phosphorylation in aged mice treated with GBP

**FIGURE 8:**
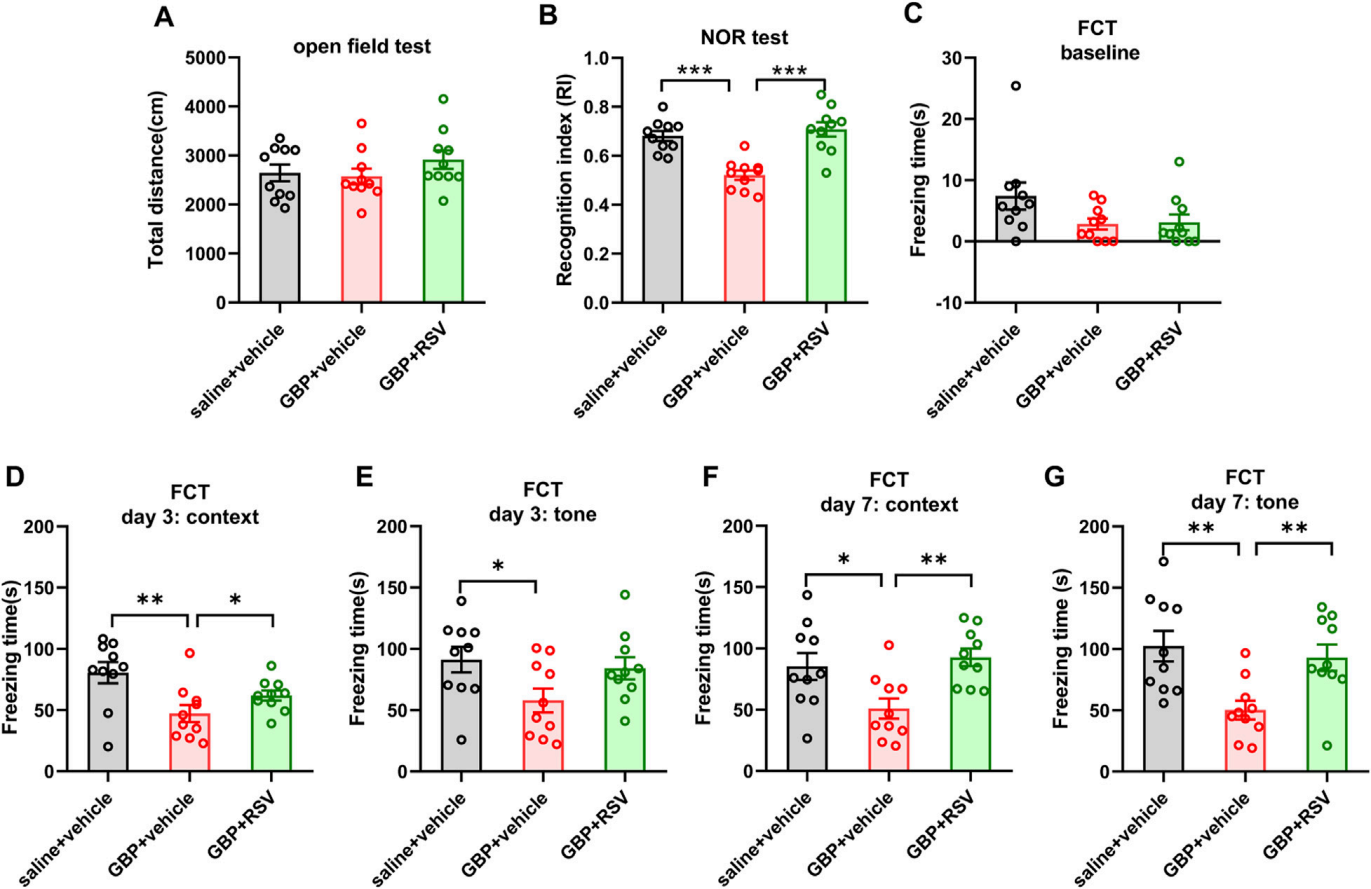
Resveratrol (RSV) prevented GBP-induced cognitive impairment in aged SNI mice. **(A)** In open field test, the total distance traveled by each of the three groups of mice was similar (one-way ANOVA, p = 0.36). **(B)** In NOR test, RSV treatment increased recognition index (RI) for a novel object in GBP-treated mice (***p < 0.001). **(C–G)** Contextual and cued fear conditioning test (FCT). **(C)** In training phase of FCT, baseline freezing time was not significantly different among three groups of mice (one-way ANOVA, p = 0.09). **(D–G)** Testing phase of FCT: tested on day 3 and 7 after training. GBP + vehicle-treated mice had shorter freezing time than saline + vehicle-treated mice in both context and tone test. For GBP treatment, mice treated with RSV had longer freezing time than mice treated with vehicle in context test and tone test. (*p < 0.05, **p < 0.01). (n = 10/group).

**FIGURE 9:**
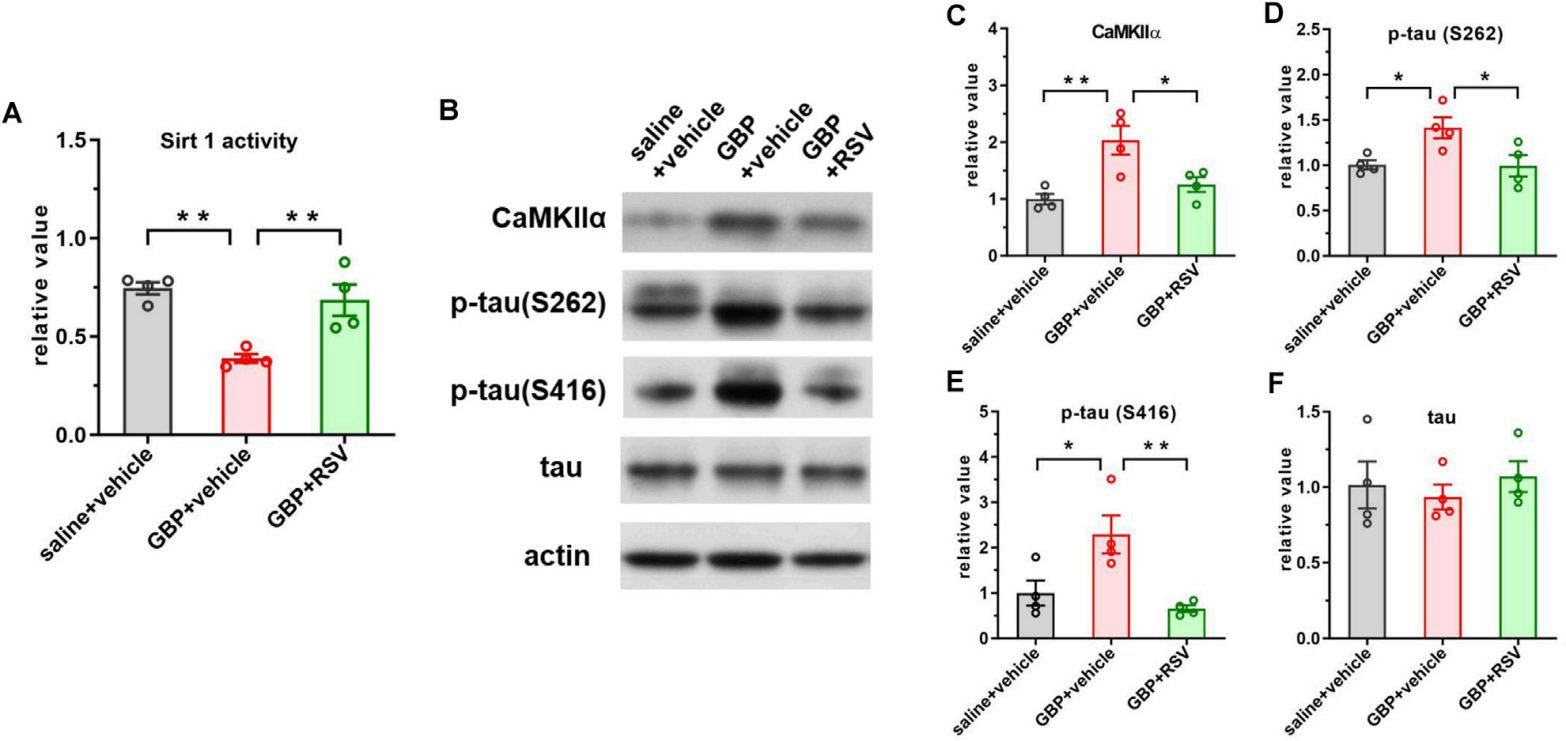
Resveratrol treatment boosted Sirt1 activity and inhibited CaMKIIα expression and tau hyperphosphorylation in GBP-treated aged SNI mice. **(A)** GBP treatment inhibited Sirt1 activity and RSV treatment restored Sirt1 activity in mice. **(B)** Western blot of CaMKIIα and tau proteins. **(C–F)** Activation of Sirt1 suppressed CaMKIIα expression, phosphorylation of p-tau (S262) and p-tau (416) in GBP-treated mice. Total tau level was not affected by resveratrol treatment. (*p < 0.05, **p < 0.01, n = 4/group).

To examine if enhancing Sirt1 activity could improve GBP-induced cogntive impairment, we treated mice with resveratrol, a Sirt1 activator. After SNI, mice were co-administered with GBP (100 mg/kg, i.p.) and resveratrol (RSV) (40 mg/kg, i.p.) for 60 days. Three groups of mice were included: saline + vehicle, GBP + vehicle, and GBP + RSV. Behaviral test was conducted as depicted in Figure 1. The dose of resveratrol was based on previous studies (Park and Pezzuto, 2015). Resveratrol treatment did not affect motor function (one-way ANOVA, p = 0.35, n = 10/group) (Figure 8A). In NOR test, resveratrol improved recognition index (RI) for the novel object in GBP-treated mice (GBP + vehicle vs. GBP + RSV: ***p < 0.001) (Figure 8B). In FCT, resveratrol treatment increased freezing time in GBP-treated mice in context and tone tests. Thus, resveratrol prevented cognitive impairments in GBP-treated mice (Figures 8A–H) (Supplementary Figures S2A,D, saline/vehicle and GBP/RSV: pre-context vs. pre-tone, ,**p < 0.01, *p < 0.05). Analysis of the hippocamal tissue indicated that reservatrol treatment restored Sirt1 activity (Figure 9A), inhibited CaMKIIa expression and hyperphosphoryaltion of tau (Figures 9B–F) in GBP-treated mice.

## 4 Discussion

GBP therapy has been a mainstay of pain management for over two decades. The use of GBP is favorable in older adults with chronic pain conditions because these patients are more susceptible to the gastrointestinal, renal and hepatic side effects of non-steroidal anti-inflammatory drugs (NSAIDs) and acetaminophen, the commonly used analgesics (Bicket and Mao, 2015). The adverse effects of GBP on brain function, including confusion, disorientation (“foggy brain”) and memory deficit, have been documented in clinical studies (Mao and Chen, 2000; Bonnet and Scherbaum, 2017; Mersfelder and Nichols, 2016; Smith et al., 2012; Oh et al., 2022; Park et al., 2022; Salinsky et al., 2002). However, the molecular mechanism underlying the adverse effects of GBP on aged brain has not been elucidated. Our study found increased tau phosphorylation via Sirt1-CaMKIIα signaling pathway in the hippocampus in aged mice with GBP-induced cognitive impairment. Overexpressing Sirt1 protein or enhancing Sirt1 activity with resveratrol ameliorated cognitive impairment and suppressed tau hyperphosphorylation in GBP-treated aged mice.

GBP inhibits calcium channel activity (Hendrich et al., 2008) to exert anti-seizure and anti-nociceptive effects. GBP binds to the α2δ1 subunit of calcium channel (Gee et al., 1996) and disrupts its trafficking. The α2δ1 subunit is abundantly expressed in the brain, especially in the hippocampus (Klugbauer et al., 1999; Cole et al., 2005; Schlick et al., 2010). We did not observe significant changes in α2δ1 expression with GBP treatment (Supplementary Figure S5), whereas GBP increased tau phosphorylation levels in the hippocampus of aged mice. Among five p-tau proteins examined, the levels of p-tau (S416) and p-tau (S262) were elevated in the brain of GBP-treated aged mice, both phosphorylation sites are associated with pathophysiology of Alzheimer’s disease (AD) (Hasegawa et al., 1992; Drewes et al., 1995; Seward et al., 2013; Amar et al., 2017). Tau protein contains eighty-five potential phosphorylation sites that can be phosphorylated by different protein kinases (Simic et al., 2016; Wagner et al., 1996; Johnson and Stoothoff, 2004). CaMKIIα, a multi-functional kinase (Swulius et al., 2008), phosphorylates S262 and S416 sites on tau (Sironi et al., 1998; Yamamoto et al., 2005; Hector et al., 2020). We analyzed the hippocampal tissue and found that GBP increased the expression of CaMKIIα and its kinase activity. This is consistent with a previous report that the cellular CaMKIIα protein level affects CaMKII kinase activities (Schulman, 2004). The expression of CaMKIIα is transcriptionally regulated by Sirt1 (Zhou et al., 2020) and retinoic acid (Chen and Kelly, 1996). Sirt1, a deacetylase predominantly found in the nucleus, is highly expressed in the brain (Zakh et al., 2010). Sirt1 regulates gene expression via direct deacetylation of histones or by promoting changes in methylation of histones and deoxyribonucleic acid (Zhang and Kraus, 2010). Sirt1 suppresses the expression of CaMKIIα via deacetylation of histones at CaMKIIα promoter in the amygdala (Zhou et al., 2020) and thereby protects against emotional pain vulnerability (Zhou et al., 2020). We analyzed Sirt1 protein expression and activity and found that GBP inhibited Sirt1 expression and activity. To further examine the role of Sirt1 in GBP-induced tau phosphorylation, we modulated Sirt1 expression and activity using AAV-mediated gene expression or resveratrol, respectively. Hippocampal overexpression of Sirt1 or systemic administration of resveratrol boosted Sirt1 activity, inhibited CaMKIIα protein expression and tau phosphorylation, and thereby prevented cognitive impairment in GBP-treated aged mice. Further study to confirm that CaMKIIα activity is also reduced concurrently with decreased CaMKIIα protein levels by overexpressing Sirt1 or resveratrol treatment will reinforce this mechanistic link. Taken together, Sirt1-CaMKIIα signaling pathway could be a molecular mechanism underlying tau hyperphosphorylation associated with cognitive impairment in GBP-treated aged mice.

Using a panel of five antibodies, we have identified GBP-induced hyperphosoryaltion at two sites on tau protein in the hippocampus of aged mice. In future studies, examining other pathological changes in tau protein would improve our understanding on how GBP affects cellular activity and brain function. The changes including the phosphoryaltion levels of other sites (e.g., p-tau 181 and p-tau 217) on tau protein, expression of tau isoforms (e.g., 4R), and acetylation of tau, should be evaluated in the hippocmapus and other brain regions. Acetylation is a pathogenic post-translational modification of tau found in the brains of AD and tauopathies (Irwin et al., 2013; Irwin et al., 2012). Sirt1 deacetylates tau, thereby reducing pathogenic tau in mouse models of tauopathy (Herskovits and Guarente, 2014) and brain injury (Shin et al., 2021). Importantly, our study highlights the role of Sirt1 in GBP-induced tau phosphorylation. How GBP regulates the expression of Sirt1 remains to be investigated, although a number of studies have shown that GBP affects gene expression in the brain (Yu et al., 2019; Alsanie et al., 2022) and cultured cells (Heo et al., 2013).

Chronic pain is associated with cognitive decline. Recently, Guerreiro *et al.* showed that chronic pain causes tau-mediated hippocampal pathology and memory deficits in adult mice (Guerreiro et al., 2022). In our study, we found that GBP-induced cognitive impairment and brain pathology are profound in aged mice, whereas SNI-induced chronic pain had a lesser impact. Several factors could contribute to the differences observed in the behavioral and pathological outcomes of SNI-induced pain in mice, including the age and sex of the mice (7-month-old male (Guerreiro et al., 2022) vs. 18-month-old female mice), the duration of pain [4 months (Guerreiro et al., 2022) vs. 2 months after SNI surgery], and protocols used for behavioral testing. Guerreiro et al. found that Rab35 (Guerreiro et al., 2022), a regulator of tau degradation, is responsible for tau pathologies. In our study, Sirt1 critically contributes to GBP-induced tau pathologies. Additionally, Guerreiro et al. showed that alleviating pain with GBP treatment appears to be protective against SNI-induced memory deficits in adult mice (Guerreiro et al., 2022). In spite of the above-mentioned differences, the two studies suggest that age is an important factor in the differential impact of pain and GBP on the brain regarding cognitive impairment and tau pathology.

Gabapentin is more frequently prescribed to women than men (Johannessen et al., 2015) as the conditions such as neuropathic pain are more prevalent among women (Ghazisaeidi et al., 2023; Mogil, 2012). Moreover, women are more likely to develop dementia and Alzheimer’s disease (Moutinho, 2025). Therefore, female mice were examined in this study. Future studies on male mice will broaden our understanding on the impact of GBP on aging brain.

In the past 10 years, the use of gabapentin has tripled in the US and it remains to be a mainstay for pain management in response to opioid crisis (Bongiovanni et al., 2023). Recent clinical studies highlight that GBP profoundly affects cognitive function in older patients (Oh et al., 2022; Park et al., 2022) and increases the risk of dementia in non-elderly patients (Eghrari et al., 2025; Huang et al., 2023). However, how GBP affects memory is understudied (Behroozi et al., 2023). Our study revealed a potential molecular mechanism underlying cognitive impairments induced by long-term GBP treatment and provided a potential remedy to curb this GBP’s adverse effect in older patients.

## Data Availability

The raw data supporting the conclusions of this article will be made available by the authors, without undue reservation.

## References

[B1] Alsanie W. F. Abdelrahman S. Alhomrani M. Gaber A. Habeeballah H. Alkhatabi H. A. (2022). Prenatal exposure to gabapentin alters the development of ventral midbrain dopaminergic neurons. Front. Pharmacol. 13, 923113. 10.3389/fphar.2022.923113 35942222 PMC9356305

[B2] Amar F. Sherman M. A. Rush T. Larson M. Boyle G. Chang L. (2017). The amyloid-β oligomer Aβ*56 induces specific alterations in neuronal signaling that lead to tau phosphorylation and aggregation. Sci. Signal. 10 (478), eaal2021. 10.1126/scisignal.aal2021 28487416 PMC5859319

[B3] Aydin O. N. Ek R. O. Temocin S. Ugur B. Alacam B. Sen S. (2012). The antinociceptive effects of systemic administration of tramadol, gabapentin and their combination on mice model of acute pain. Agri. 24 (2), 49–55. 10.5505/agri.2012.31032 22865488

[B4] Behroozi Z. Jafarpour M. Razmgir M. Saffarpour S. Azizi H. Kheirandish A. (2023). The effect of gabapentin and pregabalin administration on memory in clinical and preclinical studies: a meta-analysis and systematic review. BMC Psychiatry 23 (1), 262. 10.1186/s12888-023-04696-x 37069609 PMC10111701

[B5] Bicket M. C. Mao J. (2015). Chronic pain in older adults. Anesthesiol. Clin. 33 (3), 577–590. 10.1016/j.anclin.2015.05.011 26315639

[B6] Binder L. I. Frankfurter A. Rebhun L. I. (1985). The distribution of tau in the mammalian central nervous system. J. Cell Biol. 101 (4), 1371–1378. 10.1083/jcb.101.4.1371 3930508 PMC2113928

[B7] Bongiovanni T. Gan S. Finlayson E. Ross J. S. Harrison J. D. Boscardin W. J. (2023). Trends in the use of gabapentinoids and opioids in the postoperative period among older adults. JAMA Netw. Open 6 (6), e2318626. 10.1001/jamanetworkopen.2023.18626 37326989 PMC10276300

[B8] Bonnet U. Scherbaum N. (2017). How addictive are gabapentin and pregabalin? A systematic review. Eur. Neuropsychopharmacol. 27 (12), 1185–1215. 10.1016/j.euroneuro.2017.08.430 28988943

[B9] Celikyurt I. K. Mutlu O. Ulak G. Akar F. Y. Erden F. (2011). Gabapentin, a GABA analogue, enhances cognitive performance in mice. Neurosci. Lett. 492 (2), 124–128. 10.1016/j.neulet.2011.01.072 21296127

[B10] Chen J. Kelly P. T. (1996). Retinoic acid stimulates alpha-CAMKII gene expression in PC12 cells at a distinct transcription initiation site. J. Neurosci. 16 (18), 5704–5714. 10.1523/JNEUROSCI.16-18-05704.1996 8795626 PMC6578957

[B11] Cheng J. K. Chiou L. C. (2006). Mechanisms of the antinociceptive action of gabapentin. J. Pharmacol. Sci. 100 (5), 471–486. 10.1254/jphs.cr0050020 16474201

[B12] Cohen S. J. Stackman R. W. Jr (2015). Assessing rodent hippocampal involvement in the novel object recognition task. A review. Behav. Brain Res. 285, 105–117. 10.1016/j.bbr.2014.08.002 25169255 PMC7008635

[B13] Cole R. L. Lechner S. M. Williams M. E. Prodanovich P. Bleicher L. Varney M. A. (2005). Differential distribution of voltage-gated calcium channel alpha-2 delta (alpha2delta) subunit mRNA-containing cells in the rat central nervous system and the dorsal root ganglia. J. Comp. Neurology 491 (3), 246–269. 10.1002/cne.20693 16134135

[B14] Decosterd I. Woolf C. J. (2000). Spared nerve injury: an animal model of persistent peripheral neuropathic pain. Pain 87 (2), 149–158. 10.1016/s0304-3959(00)00276-1 10924808

[B15] Drewes G. Trinczek B. Illenberger S. Biernat J. Schmitt-Ulms G. Meyer H. E. (1995). Microtubule-associated protein/microtubule affinity-regulating kinase (p110mark). A novel protein kinase that regulates tau-microtubule interactions and dynamic instability by phosphorylation at the Alzheimer-specific site serine 262. J. Biol. Chem. 270 (13), 7679–7688. 10.1074/jbc.270.13.7679 7706316

[B16] Eghrari N. B. Yazji I. H. Yavari B. Van Acker G. M. Kim C. H. (2025). Risk of dementia following gabapentin prescription in chronic low back pain patients. Reg. Anesth. Pain Med. 2025, 106577. 10.1136/rapm-2025-106577 40639955

[B17] Eroglu C. Allen N. J. Susman M. W. O'Rourke N. A. Park C. Y. Ozkan E. (2009). Gabapentin receptor alpha2delta-1 is a neuronal thrombospondin receptor responsible for excitatory CNS synaptogenesis. Cell 139 (2), 380–392. 10.1016/j.cell.2009.09.025 19818485 PMC2791798

[B18] Fleet J. L. Dixon S. N. Kuwornu P. J. Dev V. K. Montero-Odasso M. Burneo J. (2018). Gabapentin dose and the 30-day risk of altered mental status in older adults: a retrospective population-based study. PLoS One 13 (3), e0193134. 10.1371/journal.pone.0193134 29538407 PMC5851574

[B19] Gee N. S. Brown J. P. Dissanayake V. U. Offord J. Thurlow R. Woodruff G. N. (1996). The novel anticonvulsant drug, gabapentin (Neurontin), binds to the alpha2delta subunit of a calcium channel. J. Biol. Chem. 271 (10), 5768–5776. 10.1074/jbc.271.10.5768 8621444

[B20] Ghazisaeidi S. Muley M. M. Salter M. W. (2023). Neuropathic pain: mechanisms, sex differences, and potential therapies for a global problem. Annu. Rev. Pharmacol. Toxicol. 63, 565–583. 10.1146/annurev-pharmtox-051421-112259 36662582

[B21] Gregoire S. Michaud V. Chapuy E. Eschalier A. Ardid D. (2012). Study of emotional and cognitive impairments in mononeuropathic rats: effect of duloxetine and gabapentin. Pain 153 (8), 1657–1663. 10.1016/j.pain.2012.04.023 22664319

[B22] Guerreiro S. R. Guimaraes M. R. Silva J. M. Dioli C. Vamvaka-Iakovou A. Sousa R. (2022). Chronic pain causes Tau-mediated hippocampal pathology and memory deficits. Mol. Psychiatry 27 (11), 4385–4393. 10.1038/s41380-022-01707-3 36056171

[B23] Hasegawa M. Morishima-Kawashima M. Takio K. Suzuki M. Titani K. Ihara Y. (1992). Protein sequence and mass spectrometric analyses of tau in the Alzheimer's disease brain. J. Biol. Chem. 267 (24), 17047–17054. 10.1016/s0021-9258(18)41890-x 1512244

[B24] Hayashida K. Obata H. Nakajima K. Eisenach J. C. (2008). Gabapentin acts within the locus coeruleus to alleviate neuropathic pain. Anesthesiology 109 (6), 1077–1084. 10.1097/ALN.0b013e31818dac9c 19034104 PMC2843419

[B25] Hector A. McAnulty C. Piche-Lemieux M. E. Alves-Pires C. Buee-Scherrer V. Buee L. (2020). Tau hyperphosphorylation induced by the anesthetic agent ketamine/xylazine involved the calmodulin-dependent protein kinase II. FASEB J. 34 (2), 2968–2977. 10.1096/fj.201902135R 31908108

[B26] Hendrich J. Van Minh A. T. Heblich F. Nieto-Rostro M. Watschinger K. Striessnig J. (2008). Pharmacological disruption of calcium channel trafficking by the alpha2delta ligand gabapentin. Proc. Natl. Acad. Sci. U. S. A. 105 (9), 3628–3633. 10.1073/pnas.0708930105 18299583 PMC2265195

[B27] Heo J. H. Lee S. H. Chang K. H. Han E. H. Lee S. G. Choi D. W. (2013). Identification of differentially expressed genes by gabapentin in cultured dorsal root ganglion in a rat neuropathic pain model. Biomol. Ther. Seoul. 21 (2), 126–131. 10.4062/biomolther.2013.014 24009870 PMC3762310

[B28] Herskovits A. Z. Guarente L. (2014). SIRT1 in neurodevelopment and brain senescence. Neuron 81 (3), 471–483. 10.1016/j.neuron.2014.01.028 24507186 PMC4040287

[B29] Huang Y. H. Pan M. H. Yang H. I. (2023). The association between gabapentin or pregabalin use and the risk of dementia: an analysis of the national Health insurance research database in Taiwan. Front. Pharmacol. 14, 1128601. 10.3389/fphar.2023.1128601 37324474 PMC10266423

[B30] Irwin D. J. Cohen T. J. Grossman M. Arnold S. E. Xie S. X. Lee V. M. (2012). Acetylated tau, a novel pathological signature in Alzheimer's disease and other tauopathies. Brain 135 (Pt 3), 807–818. 10.1093/brain/aws013 22366796 PMC3286338

[B31] Irwin D. J. Cohen T. J. Grossman M. Arnold S. E. McCarty-Wood E. Van Deerlin V. M. (2013). Acetylated tau neuropathology in sporadic and hereditary tauopathies. Am. J. Pathol. 183 (2), 344–351. 10.1016/j.ajpath.2013.04.025 23885714 PMC3730769

[B32] Jameson L. Frey T. Zeeberg B. Dalldorf F. Caplow M. (1980). Inhibition of microtubule assembly by phosphorylation of microtubule-associated proteins. Biochemistry 19 (11), 2472–2479. 10.1021/bi00552a027 7387985

[B33] Johannessen L. C. Beiske G. Baftiu A. Burns M. L. Johannessen S. I. (2015). Experience from therapeutic drug monitoring and gender aspects of gabapentin and pregabalin in clinical practice. Seizure 28, 88–91. 10.1016/j.seizure.2015.02.017 25758302

[B34] Johansen M. E. Maust D. T. (2024). Update to gabapentinoid use in the United States, 2002-2021. Ann. Fam. Med. 22 (1), 45–49. 10.1370/afm.3052 38253511 PMC11233090

[B35] Johnson G. V. Stoothoff W. H. (2004). Tau phosphorylation in neuronal cell function and dysfunction. J. Cell Sci. 117 (Pt 24), 5721–5729. 10.1242/jcs.01558 15537830

[B36] Klugbauer N. Lacinova L. Marais E. Hobom M. Hofmann F. (1999). Molecular diversity of the calcium channel α_2_δ subunit. J. Neurosci. 19 (2), 684–691. 10.1523/jneurosci.19-02-00684.1999 9880589 PMC6782206

[B37] Kraeuter A. K. Guest P. C. Sarnyai Z. (2019). The open field test for measuring locomotor activity and anxiety-like behavior. Methods Mol. Biol. 1916, 99–103. 10.1007/978-1-4939-8994-2_9 30535687

[B38] Kusunose N. Koyanagi S. Hamamura K. Matsunaga N. Yoshida M. Uchida T. (2010). Molecular basis for the dosing time-dependency of anti-allodynic effects of gabapentin in a mouse model of neuropathic pain. Mol. Pain 6, 83. 10.1186/1744-8069-6-83 21108841 PMC3009974

[B39] Leger M. Quiedeville A. Bouet V. Haelewyn B. Boulouard M. Schumann-Bard P. (2013). Object recognition test in mice. Nat. Protoc. 8 (12), 2531–2537. 10.1038/nprot.2013.155 24263092

[B40] Mandelkow E. M. Mandelkow E. (2012). Biochemistry and cell biology of tau protein in neurofibrillary degeneration. Cold Spring Harb. Perspect. Med. 2 (7), a006247. 10.1101/cshperspect.a006247 22762014 PMC3385935

[B41] Mao J. Chen L. L. (2000). Gabapentin in pain management. Anesth. Analg. 91 (3), 680–687. 10.1097/00000539-200009000-00034 10960399

[B42] Mersfelder T. L. Nichols W. H. (2016). Gabapentin: abuse, dependence, and withdrawal. Ann. Pharmacother. 50 (3), 229–233. 10.1177/1060028015620800 26721643

[B43] Mogil J. S. (2012). Sex differences in pain and pain inhibition: multiple explanations of a controversial phenomenon. Nat. Rev. Neurosci. 13 (12), 859–866. 10.1038/nrn3360 23165262

[B44] Moore R. A. Wiffen P. J. Derry S. Toelle T. Rice A. S. (2014). Gabapentin for chronic neuropathic pain and fibromyalgia in adults. Cochrane Database Syst. Rev. (4), CD007938. 10.1002/14651858.CD007938.pub2 24771480 PMC6464253

[B45] Moutinho S. (2025). Women twice as likely to develop Alzheimer's disease as men - but scientists do not know why. Nat. Med. 31 (3), 704–707. 10.1038/s41591-025-03564-3 40087515

[B46] Oh G. Moga D. C. Fardo D. W. Abner E. L. (2022). The association of gabapentin initiation and neurocognitive changes in older adults with normal cognition. Front. Pharmacol. 13, 910719. 10.3389/fphar.2022.910719 36506564 PMC9732650

[B47] Park E. J. Pezzuto J. M. (2015). The pharmacology of resveratrol in animals and humans. Biochim. Biophys. Acta 1852 (6), 1071–1113. 10.1016/j.bbadis.2015.01.014 25652123

[B48] Park C. M. Inouye S. K. Marcantonio E. R. Metzger E. Bateman B. T. Lie J. J. (2022). Perioperative gabapentin use and in-hospital adverse clinical events among older adults after major surgery. JAMA Intern Med. 182 (11), 1117–1127. 10.1001/jamainternmed.2022.3680 36121671 PMC9486639

[B49] Pauly N. J. Delcher C. Slavova S. Lindahl E. Talbert J. Freeman P. R. (2020). Trends in gabapentin prescribing in a commercially Insured U.S. Adult population, 2009-2016. J. Manag. Care Specialty Pharm. 26 (3), 246–252. 10.18553/jmcp.2020.26.3.246 32105169 PMC7155217

[B50] Salinsky M. C. Binder L. M. Oken B. S. Storzbach D. Aron C. R. Dodrill C. B. (2002). Effects of gabapentin and carbamazepine on the EEG and cognition in healthy volunteers. Epilepsia 43 (5), 482–490. 10.1046/j.1528-1157.2002.22501.x 12027908

[B51] Schlick B. Flucher B. E. Obermair G. J. (2010). Voltage-activated calcium channel expression profiles in mouse brain and cultured hippocampal neurons. Neuroscience 167 (3), 786–798. 10.1016/j.neuroscience.2010.02.037 20188150 PMC3315124

[B52] Schneider C. A. Rasband W. S. Eliceiri K. W. (2012). NIH Image to ImageJ: 25 years of image analysis. Nat. Methods 9 (7), 671–675. 10.1038/nmeth.2089 22930834 PMC5554542

[B53] Schulman H. (2004). Activity-dependent regulation of calcium/calmodulin-dependent protein kinase II localization. J. Neurosci. 24 (39), 8399–8403. 10.1523/JNEUROSCI.3606-04.2004 15456811 PMC6729891

[B54] Seward M. E. Swanson E. Norambuena A. Reimann A. Cochran J. N. Li R. (2013). Amyloid-beta signals through tau to drive ectopic neuronal cell cycle re-entry in Alzheimer's disease. J. Cell Sci. 126 (Pt 5), 1278–1286. 10.1242/jcs.1125880 23345405 PMC3635465

[B55] Shin M. K. Vazquez-Rosa E. Koh Y. Dhar M. Chaubey K. Cintron-Perez C. J. (2021). Reducing acetylated tau is neuroprotective in brain injury. Cell 184 (10), 2715–2732.e23. 10.1016/j.cell.2021.03.032 33852912 PMC8491234

[B56] Simic G. Babic Leko M. Wray S. Harrington C. Delalle I. Jovanov-Milosevic N. (2016). Tau protein hyperphosphorylation and aggregation in Alzheimer’s disease and other tauopathies, and possible neuroprotective strategies. Biomolecules 6 (1), 6. 10.3390/biom6010006 26751493 PMC4808800

[B57] Sironi J. J. Yen S. H. Gondal J. A. Wu Q. Grundke-Iqbal I. Iqbal K. (1998). Ser-262 in human recombinant tau protein is a markedly more favorable site for phosphorylation by CaMKII than PKA or PhK. FEBS Lett. 436 (3), 471–475. 10.1016/s0014-5793(98)01185-5 9801171

[B58] Smith B. H. Higgins C. Baldacchino A. Kidd B. Bannister J. (2012). Substance misuse of gabapentin. Br. J. General Pract. 62 (601), 406–407. 10.3399/bjgp12X653516 22867659 PMC3404313

[B59] Striano P. Striano S. (2008). Gabapentin: a Ca^2+^ channel alpha 2-delta ligand far beyond epilepsy therapy. Drugs Today 44 (5), 353–368. 10.1358/dot.2008.44.5.1186403 18548137

[B60] Swulius M. T. Waxham M. N. (2008). Ca(2+)/calmodulin-dependent protein kinases. Cell. Mol. Life Sci. CMLS 65 (17), 2637–2657. 10.1007/s00018-008-8086-2 18463790 PMC3617042

[B61] Wagner U. Utton M. Gallo J. M. Miller C. C. (1996). Cellular phosphorylation of tau by GSK-3 beta influences tau binding to microtubules and microtubule organisation. J. Cell Sci. 109 (Pt 6), 1537–1543. 10.1242/jcs.109.6.1537 8799840

[B62] Wang X. Zhang C. Szabo G. Sun Q. Q. (2013). Distribution of CaMKIIα expression in the brain *in vivo*, studied by CaMKIIα-GFP mice. Brain Res. 1518, 9–25. 10.1016/j.brainres.2013.04.042 23632380 PMC3747672

[B63] Yamamoto H. Hiragami Y. Murayama M. Ishizuka K. Kawahara M. Takashima A. (2005). Phosphorylation of tau at serine 416 by Ca^2+^/calmodulin-dependent protein kinase II in neuronal soma in brain. J. Neurochem. 94 (5), 1438–1447. 10.1111/j.1471-4159.2005.03307.x 16000144

[B64] Yu J. Wang D. S. Bonin R. P. Penna A. Alavian-Ghavanini A. Zurek A. A. (2019). Gabapentin increases expression of delta subunit-containing GABA(A) receptors. EBioMedicine 42, 203–213. 10.1016/j.ebiom.2019.03.008 30878595 PMC6491385

[B65] Zakhary S. M. Ayubcha D. Dileo J. N. Jose R. Leheste J. R. Horowitz J. M. (2010). Distribution analysis of deacetylase SIRT1 in rodent and human nervous systems. Anat. Rec. Hob. 293 (6), 1024–1032. 10.1002/ar.21116 20225204 PMC3071026

[B66] Zhang T. Kraus W. L. (2010). SIRT1-dependent regulation of chromatin and transcription: linking NAD(+) metabolism and signaling to the control of cellular functions. Biochim. Biophys. Acta 1804 (8), 1666–1675. 10.1016/j.bbapap.2009.10.022 19879981 PMC2886162

[B67] Zhou C. Wu Y. Ding X. Shi N. Cai Y. Pan Z. Z. (2020). SIRT1 decreases emotional pain vulnerability with associated CaMKIIα deacetylation in central amygdala. J. Neurosci. 40 (11), 2332–2342. 10.1523/JNEUROSCI.1259-19.2020 32005763 PMC7083291

