# Supplementary material for: Long-term gabapentin treatment impairs cognitive function in aged mice via tau hyperphosphorylation
Source: Front Pharmacol. 2025 Sep 3;16:1616775. doi: 10.3389/fphar.2025.1616775 (PMC12440869; doi:10.3389/fphar.2025.1616775)
Supplement: Supplementary file 1 [file DataSheet1.docx]

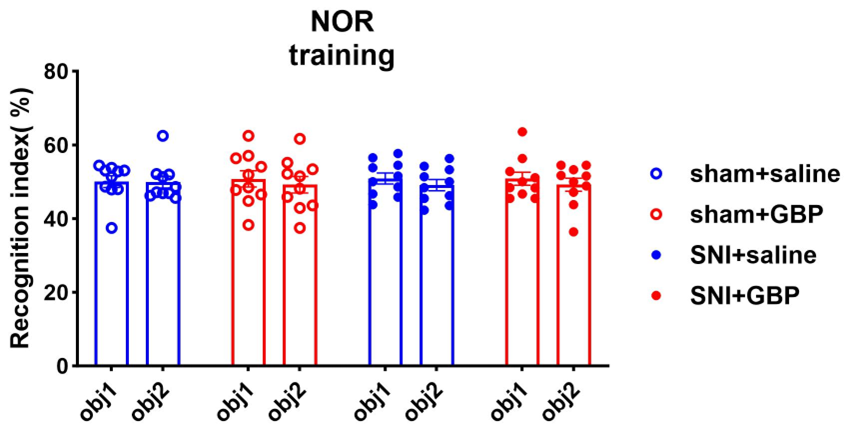
**Supplementary Figure S1. Recognition index of training session of novel object recognition test.** In training session of NOR test, the mice spent similar time exploring two identical objects (obj1 and obj2) showing similar recognition index (RI) for both objects.

**
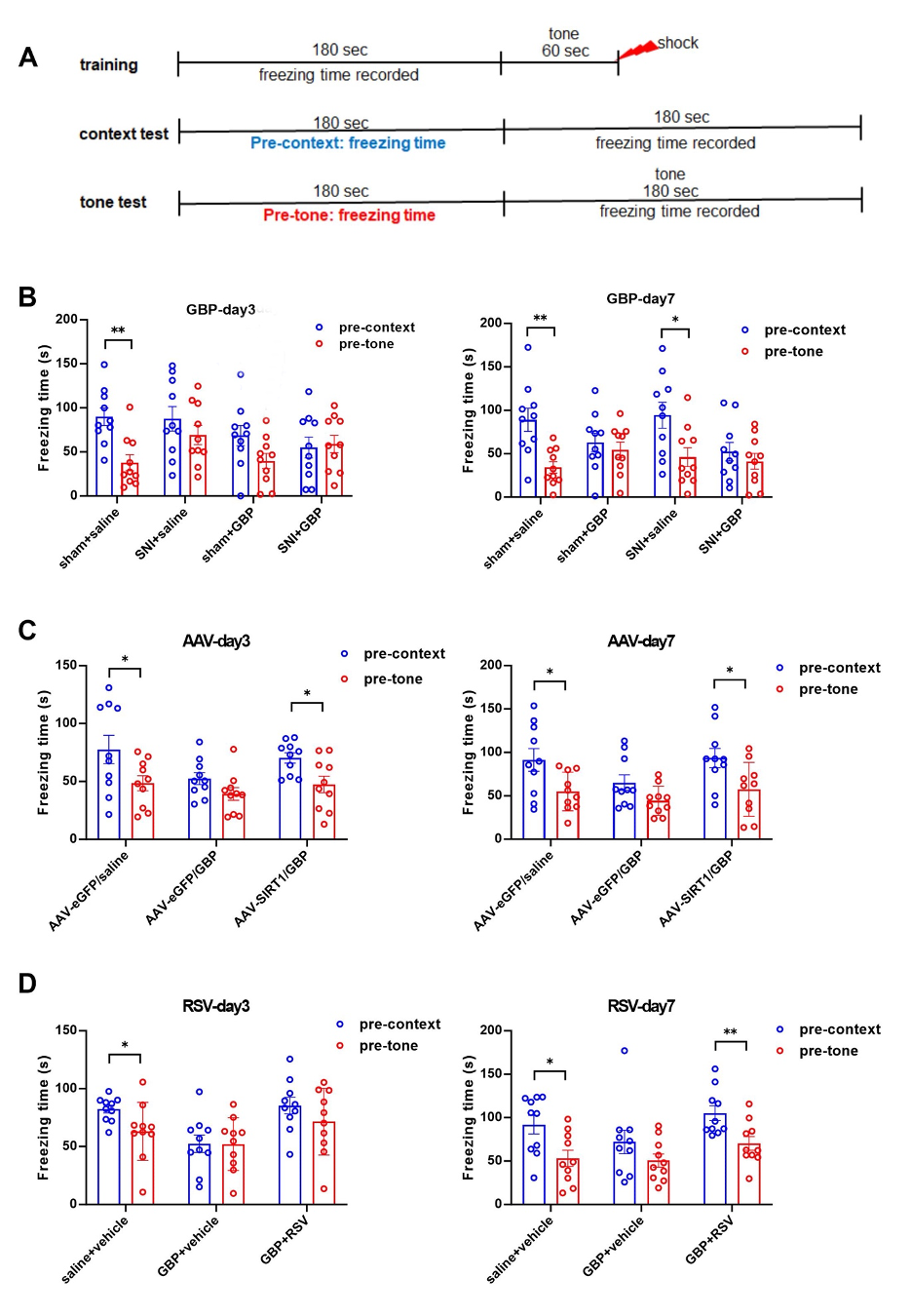
Supplementary Figure S2. GBP treatment impaired cognitive function.** Illustration of the protocol for contextual and cued fear conditioning test (FCT) (A). Pre-tone freezing time was shorter than pre-context freezing time in mice with normal cognitive function. The groups were sham/saline and SNI/saline (B), AAV-eGFP/saline and AAV-Sirt1/GBP (C), saline/vehicle and GBP/RSV (D). In groups with impaired cognitive function, the mice did not remember the environment (black and white stripe vs. checkered pattern), and therefore the freezing time did not differ between pre-context and pre-tone periods. The groups were sham/GBP and SNI/GBP (B), AAV-eGFP/GBP (C), and GBP/vehicle (D). (** p<0.01, *p<0.05, n = 10/group)

**

Supplementary Figure S3. GBP attenuated nociception in aged mice after spared nerve injury (SNI). (A)** In von Frey test, SNI mice had a significantly lowered paw withdrawal threshold than sham mice when the test was conducted prior to daily GBP (100 mg/kg, i.p.) or saline administration (sham vs. SNI: two-way ANOVA, p<0.001, n=10/group). **(B)** Assessed before and 2 hours after GBP administration, GBP effectively improved mechanical allodynia in SNI mice (day 63, before SNI+GBP vs. after SNI+GBP ***p<0.001).


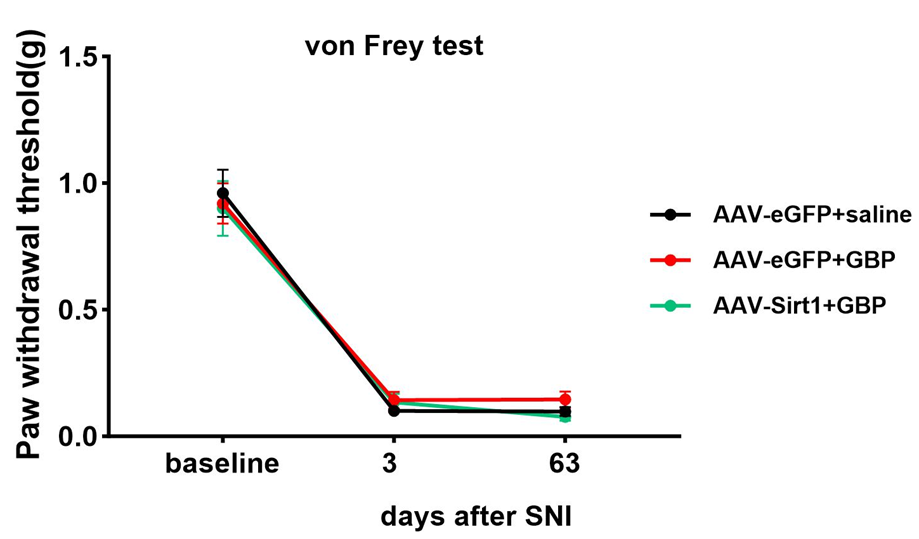


**Supplementary Figure S4. Sirt1 overexpression in the hippocampus did not affect nociception in aged mice after spared nerve injury (SNI).** von Frey test was performed at 3 weeks after AAV-Sirt1 or AAV-eGFP infusion (baseline), 3 days and 63 days after SNI. All three groups of mice developed mechanical allodynia.


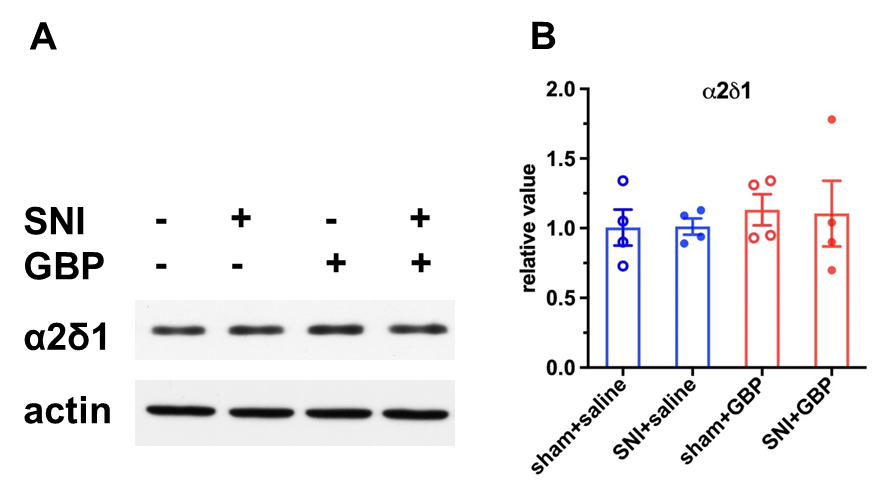
**Supplementary Figure S5. GBP treatment did not affect the expression of α2δ1 in the hippocampus.** Western blot image (**A**) and quantification (**B**) of the expression of α2δ1 in the hippocampus (one-way ANOVA, p = 0.90, n=4/group).

**Fig 4&5**


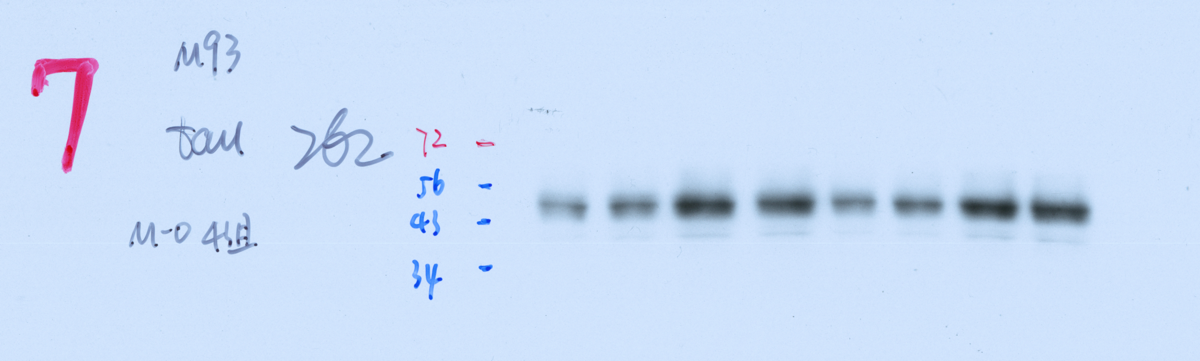


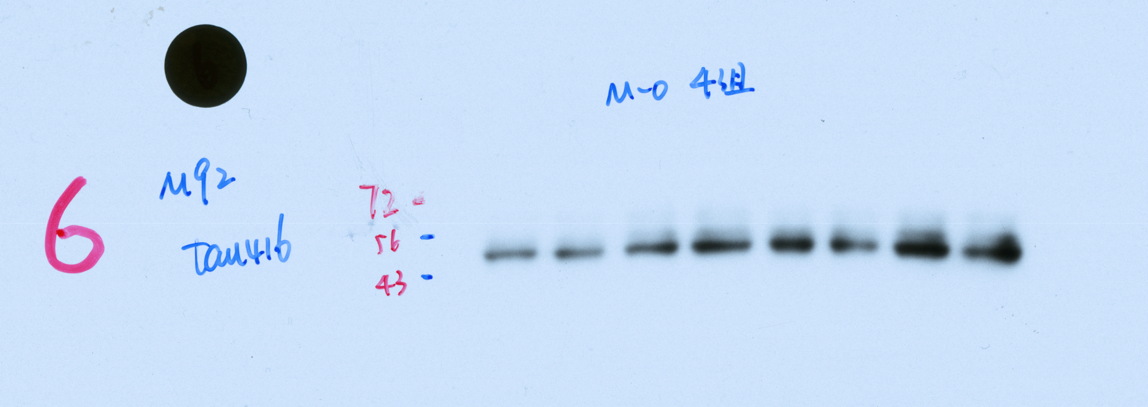


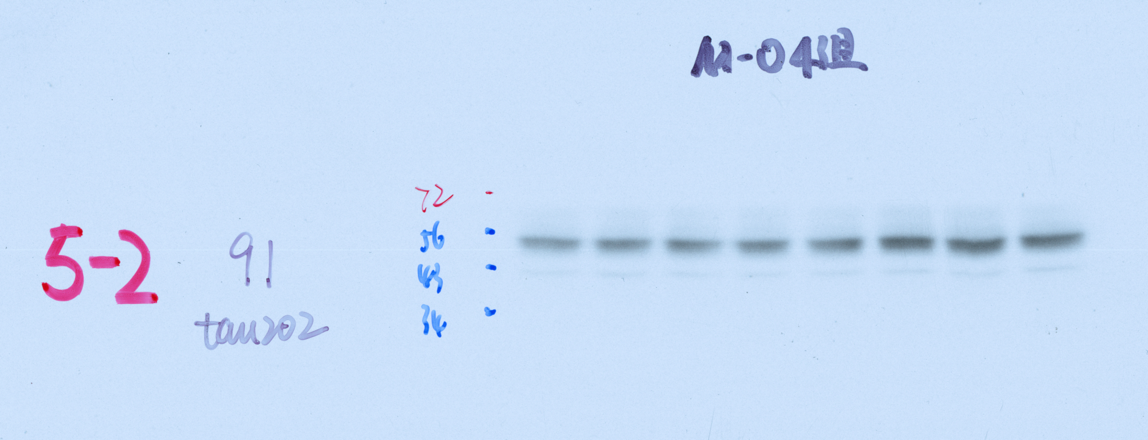


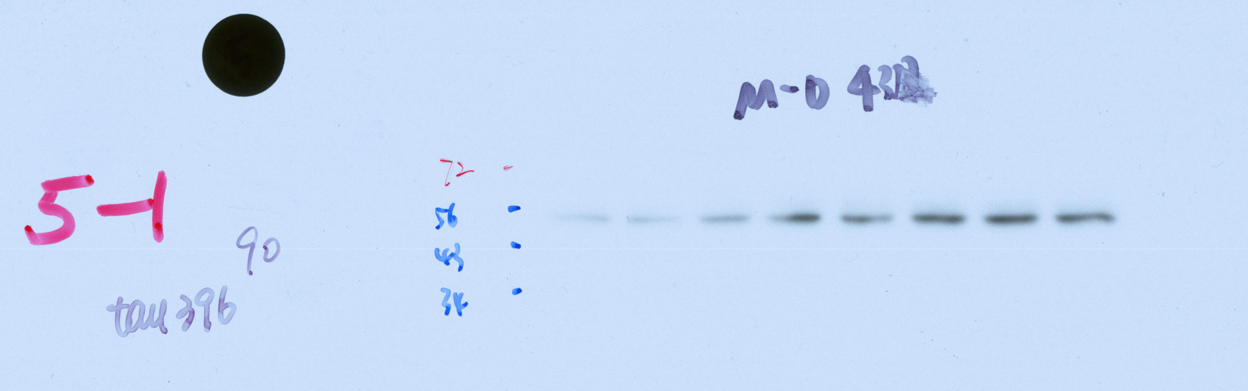


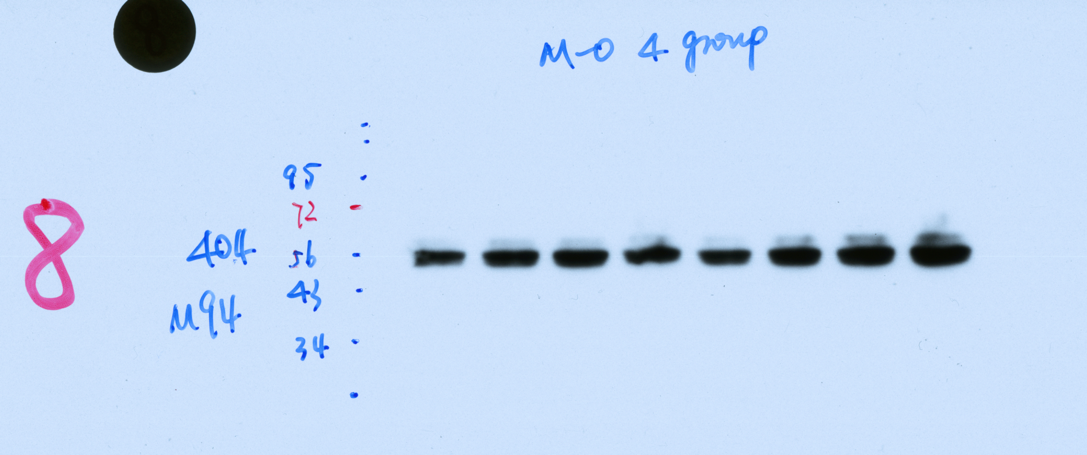


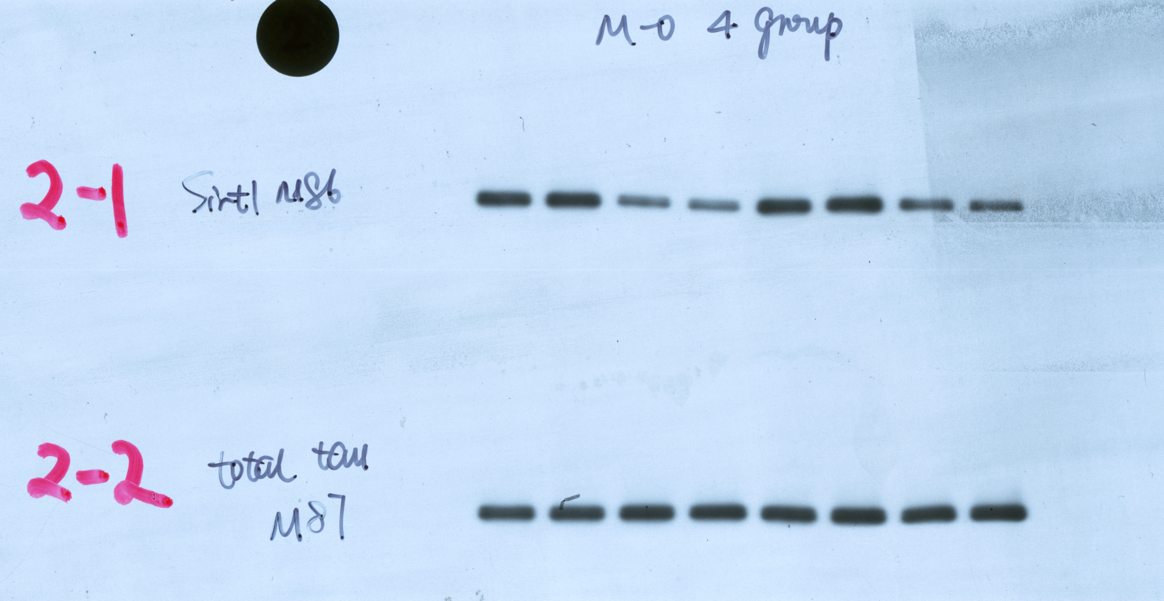


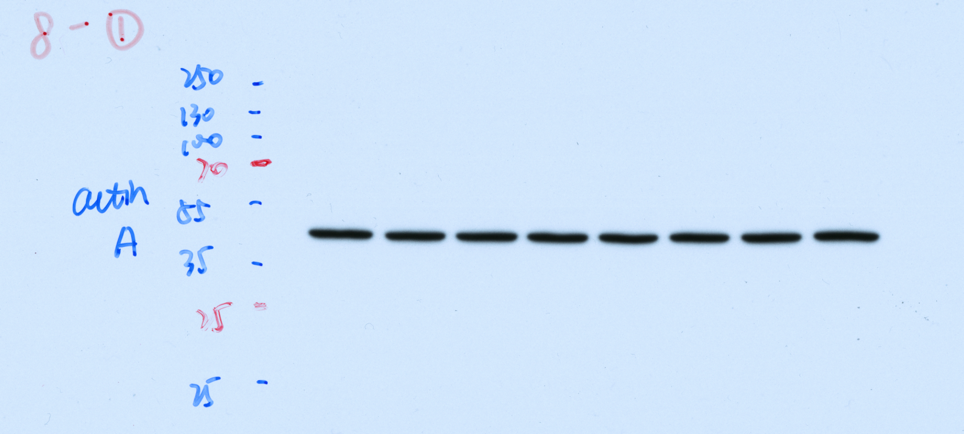


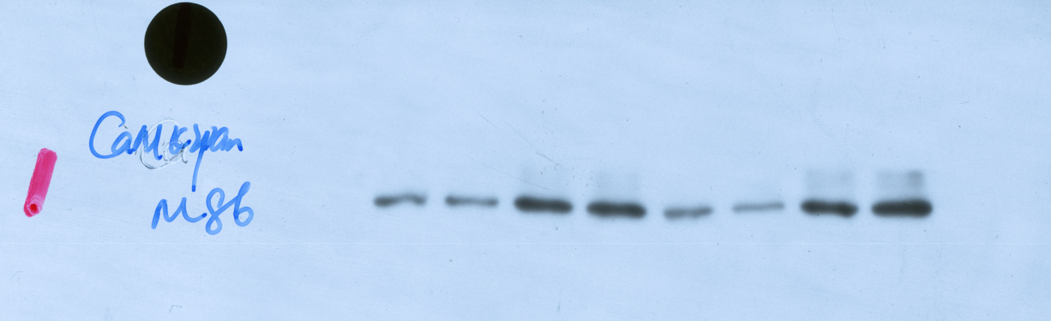


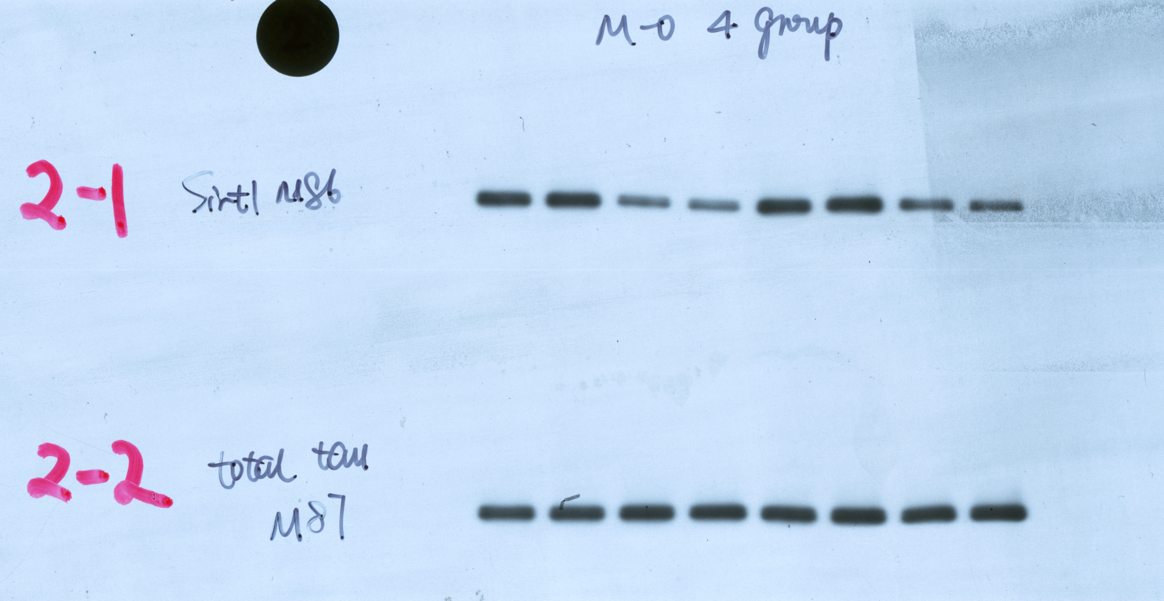


**Fig 7**


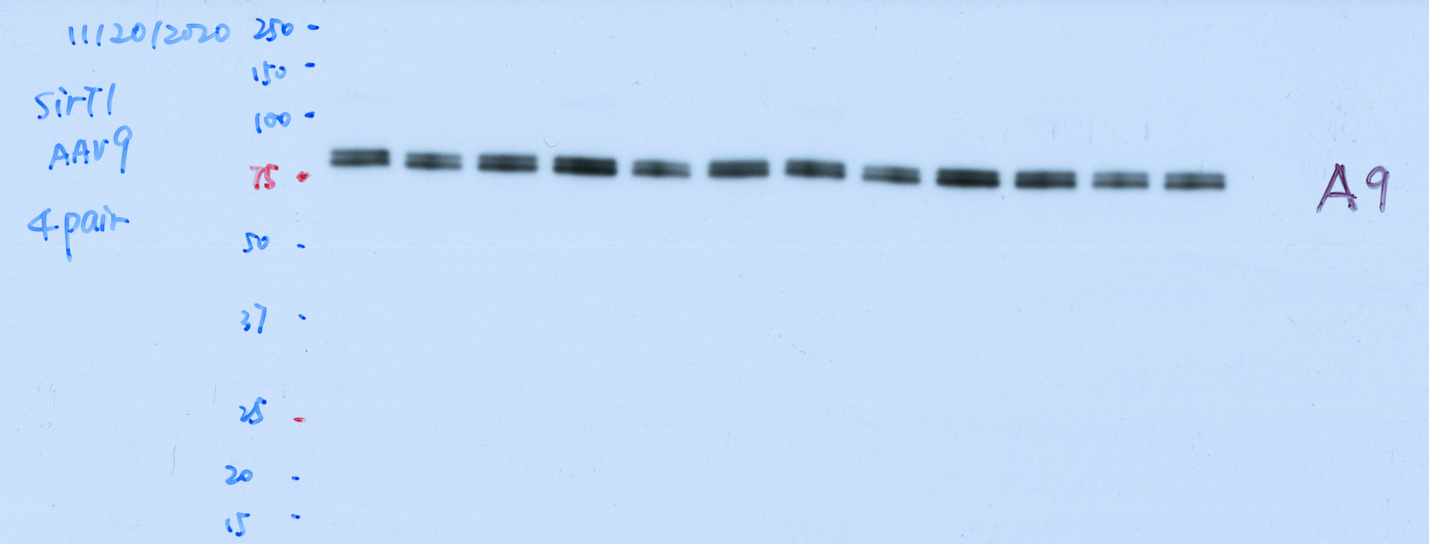


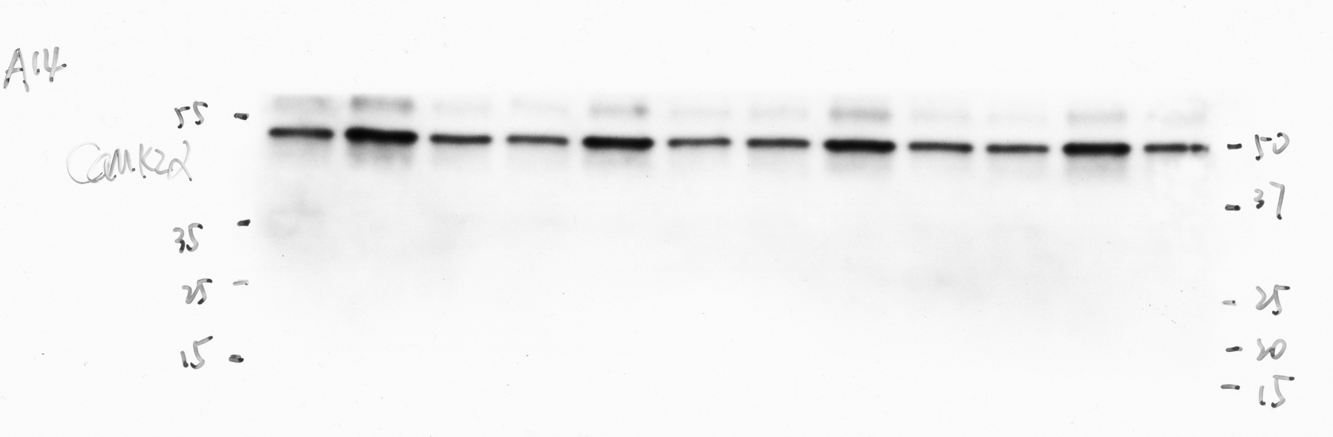

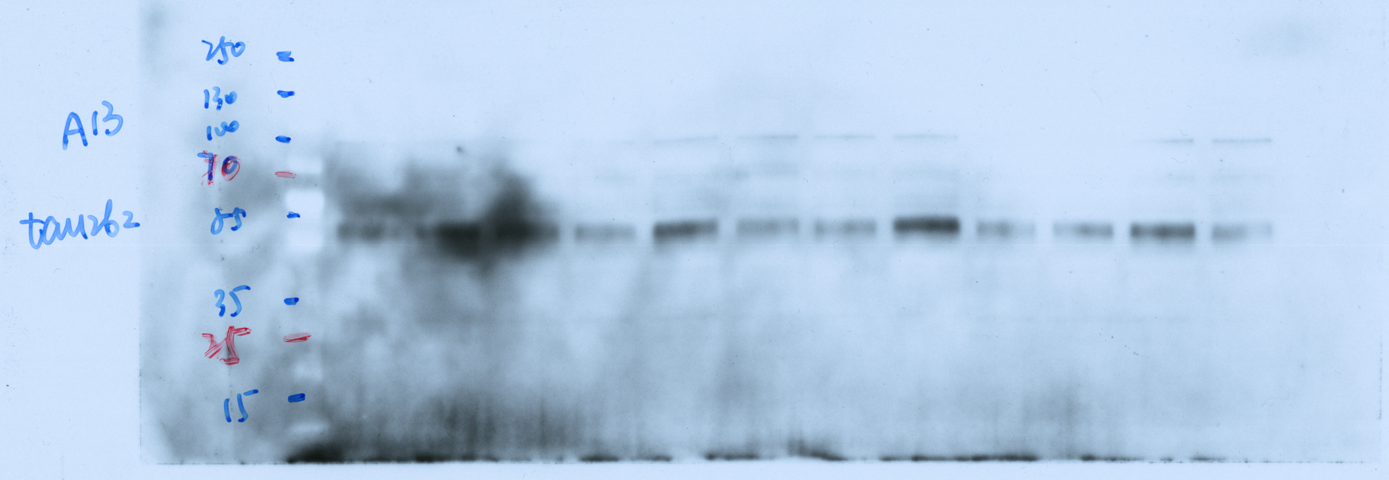


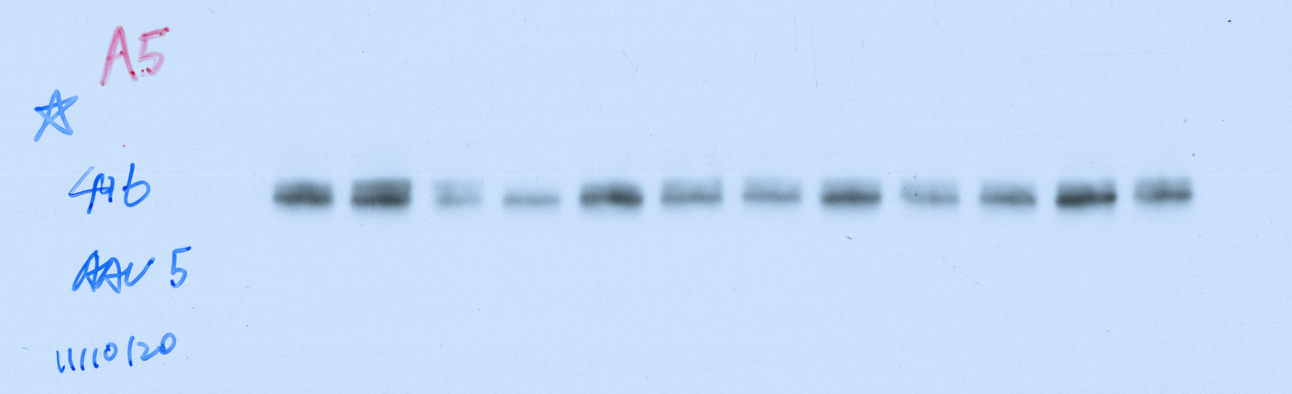


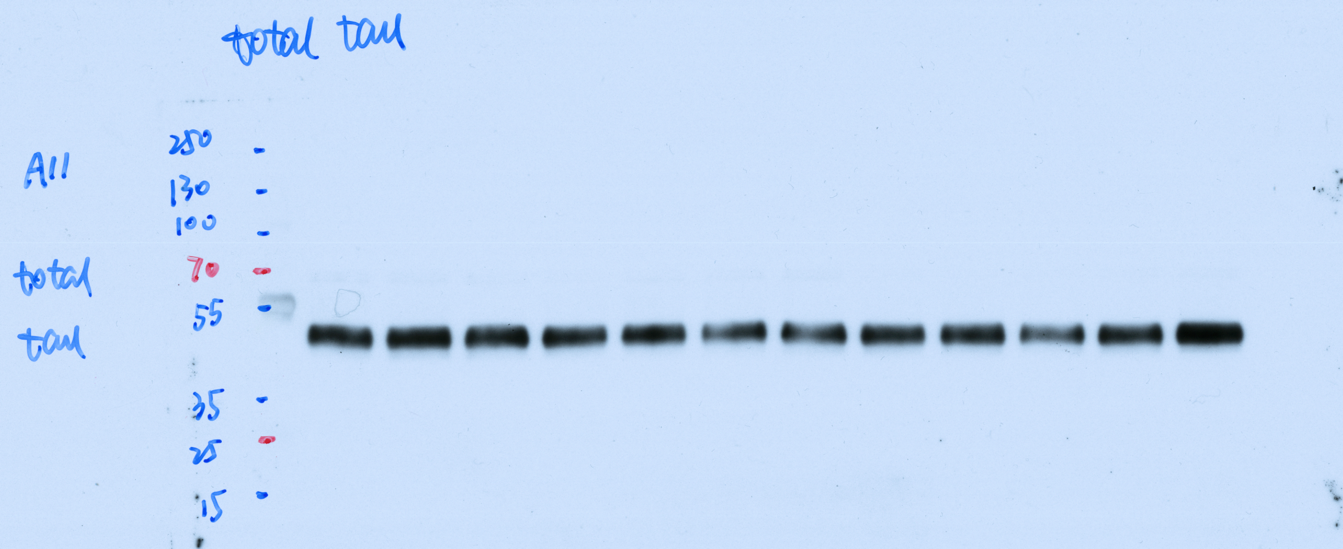


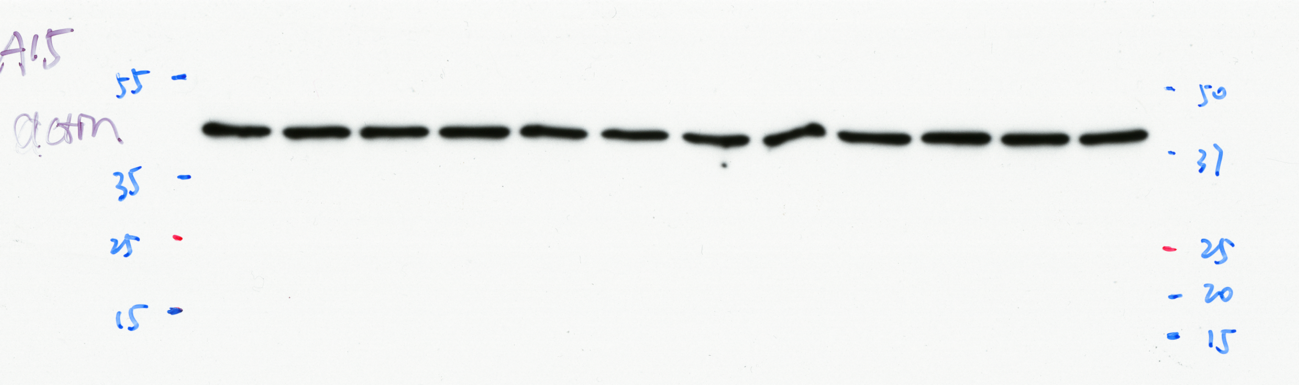


**Fig 9**

**
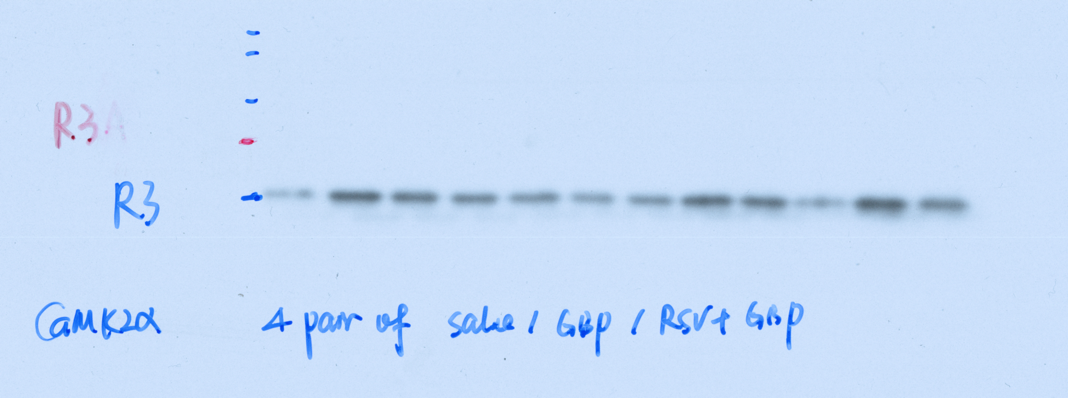
**

**
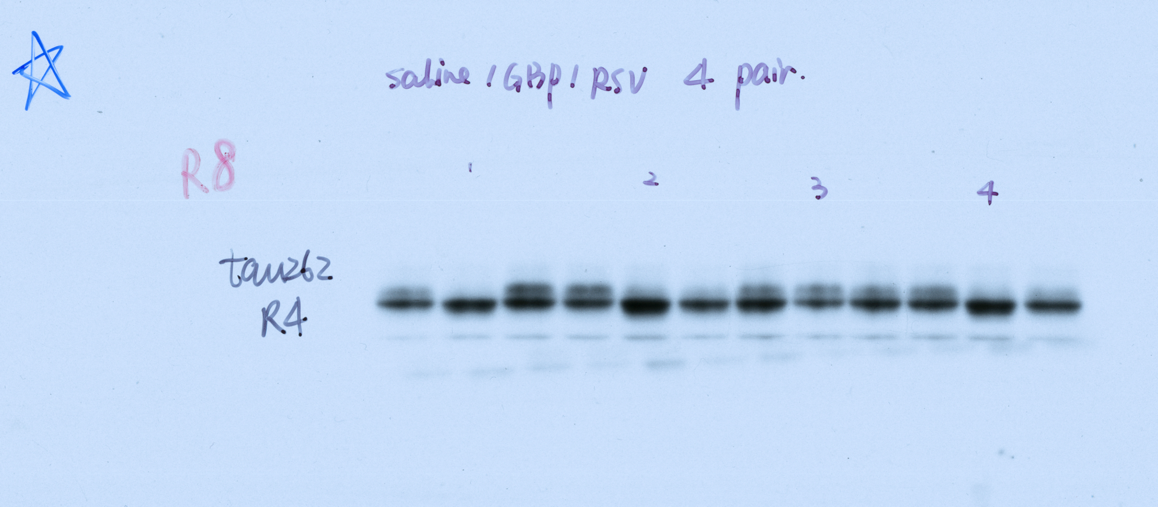
**

**
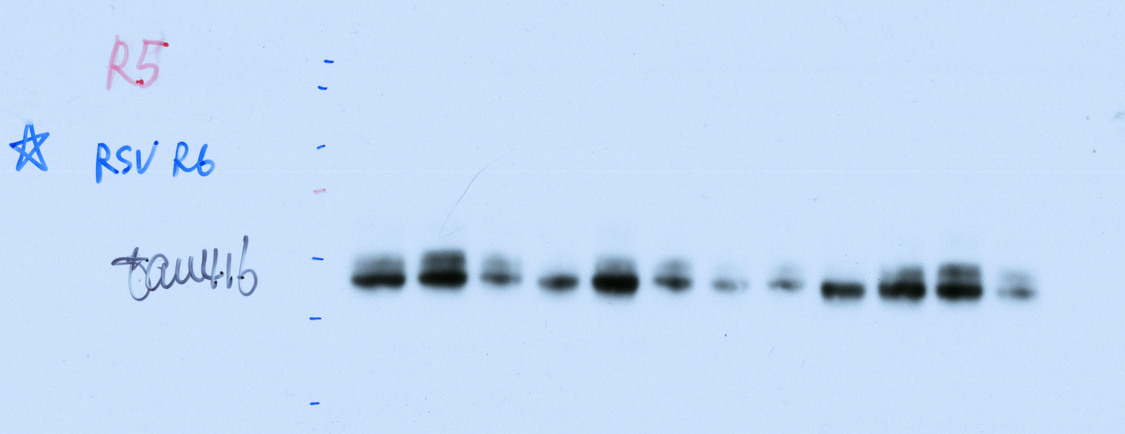
**

**
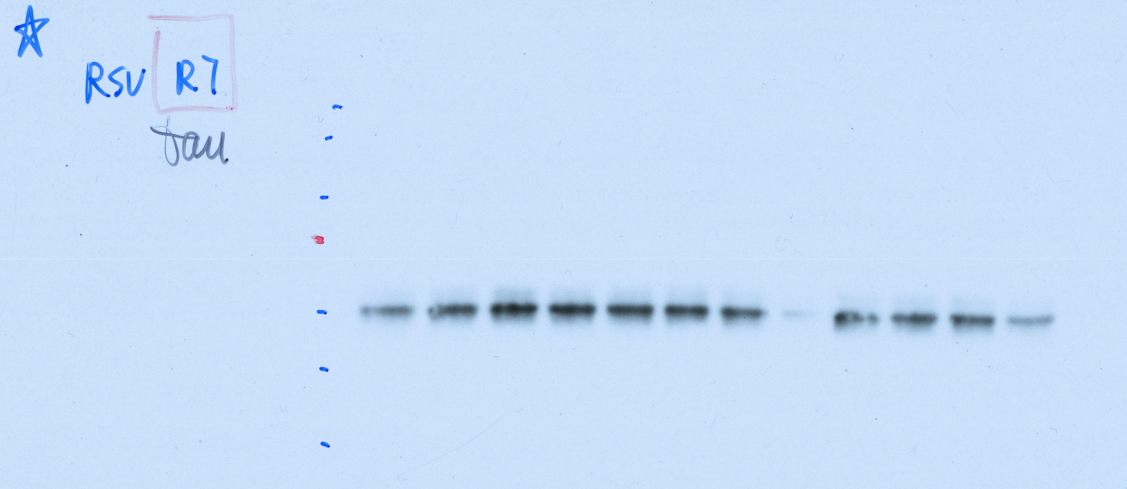
**

**
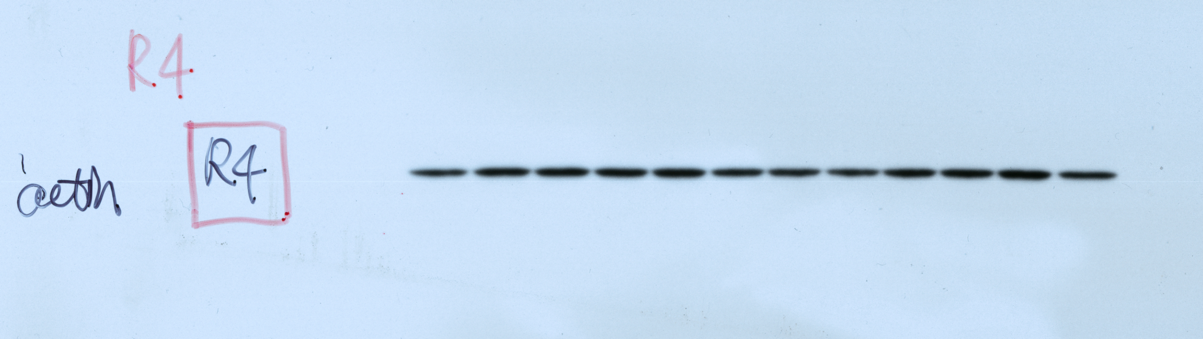
**
